# Pyroptosis and inflammasomes in cancer and inflammation

**DOI:** 10.1002/mco2.374

**Published:** 2023-09-24

**Authors:** Jie‐Lin Wang, Sheng‐Ni Hua, Hai‐Juan Bao, Jing Yuan, Yang Zhao, Shuo Chen

**Affiliations:** ^1^ Department of Obstetrics and Gynecology Guangzhou Key Laboratory of Targeted Therapy for Gynecologic Oncology Guangdong Provincial Key Laboratory of Major Obstetric Diseases The Third Affiliated Hospital of Guangzhou Medical University Guangzhou China; ^2^ Department of Gynecologic Oncology Research Office Guangzhou Key Laboratory of Targeted Therapy for Gynecologic Oncology Guangdong Provincial Key Laboratory of Major Obstetric Diseases The Third Affiliated Hospital of Guangzhou Medical University Guangzhou China; ^3^ Department of Radiation Oncology Zhuhai People s Hospital Zhuhai Hospital Affiliated with Jinan University Zhuhai China

**Keywords:** cancer, inflammasome, inflammation, pyroptosis, targeted treatment

## Abstract

Nonprogrammed cell death (NPCD) and programmed cell death (PCD) are two types of cell death. Cell death is significantly linked to tumor development, medication resistance, cancer recurrence, and metastatic dissemination. Therefore, a comprehensive understanding of cell death is essential for the treatment of cancer. Pyroptosis is a kind of PCD distinct from autophagy and apoptosis in terms of the structure and function of cells. The defining features of pyroptosis include the release of an inflammatory cascade reaction and the expulsion of lysosomes, inflammatory mediators, and other cellular substances from within the cell. Additionally, it displays variations in osmotic pressure both within and outside the cell. Pyroptosis, as evidenced by a growing body of research, is critical for controlling the development of inflammatory diseases and cancer. In this paper, we reviewed the current level of knowledge on the mechanism of pyroptosis and inflammasomes and their connection to cancer and inflammatory diseases. This article presents a theoretical framework for investigating the potential of therapeutic targets in cancer and inflammatory diseases, overcoming medication resistance, establishing nanomedicines associated with pyroptosis, and developing risk prediction models in refractory cancer. Given the link between pyroptosis and the emergence of cancer and inflammatory diseases, pyroptosis‐targeted treatments may be a cutting‐edge treatment strategy.

## Graphical abstract

Pyroptosis is characterized by cell swelling as well as the release of inflammatory mediators, which has been identified that it was induced by four distinct signaling pathways. Pyroptosis is crucial for controlling the development of cancer and inflammation diseases. It can be pro‐ or anti‐cancer. Given the link between pyroptosis and the emergence of cancer and inflammatory diseases, pyroptosis‐targeted treatments may be a cutting‐edge treatment strategy.

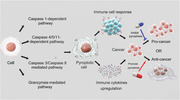

## 1 INTRODUCTION

It is vital to understand how to effectively eradicate cancer cells while protecting healthy cells in cancer treatment. Apoptosis promotion is one of the most effective methods to treat cancer. Unfortunately, the use of apoptosis in tumor therapy is restricted. According to existing studies, certain cancers exhibit resistance to chemotherapeutic treatments because of abnormal apoptosis.^1^ Hence, a strategy for fighting cancer that involves turning on other programmed cell deaths (like pyroptosis) instead of apoptosis appears promising.

Pyroptosis is a kind of PCD distinct from autophagy and apoptosis in terms of the structure and function of cells. Its defining characteristics include the release of an inflammatory cascade reaction and the expulsion of inflammatory mediators, and other cellular substances from within the cell. It also presents variations in the osmotic pressure within and outside the cell.^2^ Inflammasomes are a complex of proteins charged with initiating the inflammatory response and activating pyroptosis. The activation of inflammasomes is a crucial step in initiating the pyroptotic pathway.^3^, ^4^ In addition to causing PCD, pyroptosis and inflammasomes also regulate immune responses by promoting the maturation and secretion of proinflammatory cytokines.^5^, ^6^Furthermore, they contribute to the recruitment and activation of immune cells.^7^ In the end, they affect the tumor microenvironment (TME), triggering an inflammatory response that can be procancer or antitumor. Globally, cancer is a serious public health problem. For example, the United States is anticipated to experience 1,918,030 new cases of cancer along with 609,360 cancer‐related deaths in 2022.^8^ The pressing need for effective prevention, early identification, and treatment strategies is highlighted by these statistics. Pyroptosis can effectively eliminate cancer cells by triggering inflammation and immune responses. This emerging understanding of pyroptosis offers exciting opportunities for the development of innovative therapies and tailored treatments for various types of cancer. Understanding the mechanisms of pyroptosis and inflammasomes in cancer and inflammation is crucial for developing targeted therapies.

On the other hand, pyroptosis can trigger the release of proinflammatory cytokines, leading to an amplification of the inflammatory response.^9^, ^10^ Additionally, understanding the regulation of pyroptosis could also help in identifying potential therapeutic targets for diseases characterized by chronic inflammation. Furthermore, inflammasomes, which are multiprotein complexes, play a crucial role in triggering and regulating pyroptosis. Inflammasomes can sense danger signals and activate caspases, which then cleave and activate cytokines like interleukin‐1β (IL‐1β) and IL‐18.^3^, ^4^ Therefore, targeting inflammasomes could be another promising avenue for developing anti‐inflammatory therapies.

Roles of pyroptosis and inflammasomes in inflammatory diseases and cancer and their promise as a cutting‐edge treatment approach are covered in this review. In addition, we emphasized the necessity of comprehending the mechanisms driving pyroptosis in various types of cancer as well as some associated inhibitors, chemo drugs, and nano drugs to modulate tumor progression and immune response in the manner of pyroptosis. Moreover, we discussed the challenges and limits of targeting pyroptosis in cancer treatment. It is beneficial for the development of targeted therapies that selectively induce cancer cell death while sparing normal cells by understanding the molecular pathways behind pyroptosis.

## 2 PYROPTOSIS AND ITS MECHANISMS

### 2.1 Definition and background of pyroptosis

**FIGURE 1:**
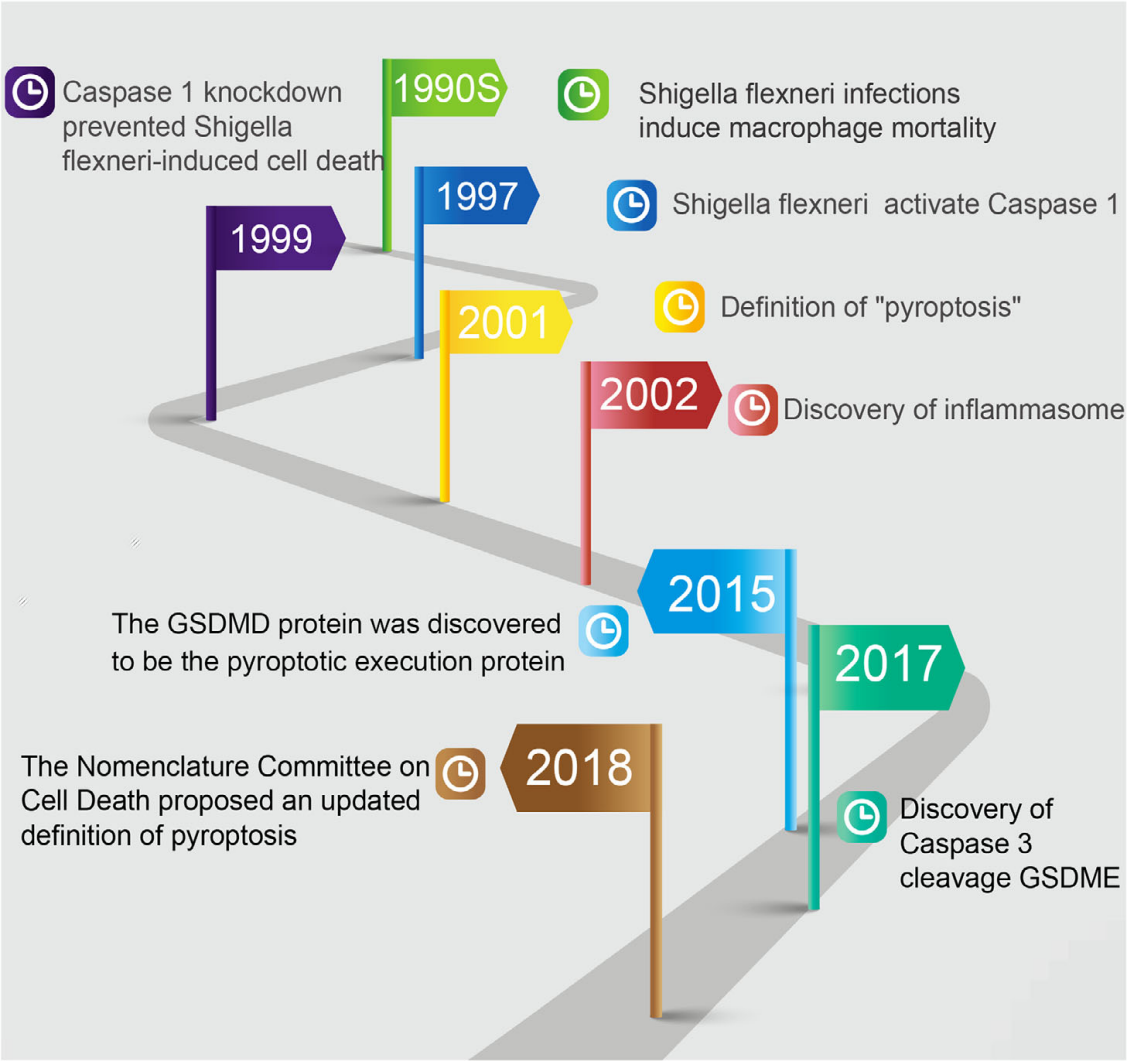
Major developments in the history of pyroptosis. The development of pyroptosis from the initial discovery, discovery of key molecules, and definition.

Pyroptosis, which combines the Greek words “pyro” (which means fire) and “ptosis” (which means failure), is a comparatively modern term for inflammatory PCD.^11^ During the 1990s, it was widely understood that Shigella flexneri infections might induce macrophage mortality in mice or humans.^2^ Shigella flexneri might activate caspase 1 in host cells based on research conducted by Zychlinsky et al. in 1997.^12^ Two years later, the scientists found that the caspase 1 knockdown prevented the cell death induced by Shigella flexneri.^13^ In 2001, Cookson demonstrated that the cell death brought on by a bacterial infection was separate from apoptosis and named it “pyroptosis^11^” Martinon et al. discovered the caspase activating complex known as the inflammasome in 2002. They noticed that the inflammasome comprised the nucleotide‐binding oligomerization domain (NOD)‐like receptor (NLR) family, pyrin domain containing 1 (NLRP1), caspase 1, and apoptosis‐associated speck‐like protein (ASC), which was engaged in caspase activation.^14^ Since then, classical inflammasomes composed of several receptors have been identified. Shi's team reported that gasdermin‐D (GSDMD) was in a state of self‐inhibition in a typical microenvironment in 2015. The action of caspase 1 degraded GSDMD into two peptides, the N‐ and C‐terminal fragments. The N‐terminal segment created an incredibly small hole in the cell membrane. The GSDMD protein was subsequently discovered to be the pyroptotic execution protein.^6^ The GSDMA, GSDMB, GSDMC, DFNA5/GSDME, and DFNB59 are all GSDM family members. Members of the GSDM family can trigger pyroptosis by generating plasma membrane pores on cell membranes.^15^ Particularly, the discovery that caspase 3 cleaved GSDME protein and induced pyroptosis in 2017 is an important turning point in the study of pyroptosis.^16^ The Nomenclature Committee on Cell Death proposed an updated definition of pyroptosis in 2018.^17^ Since that time, pyroptosis has been well defined. The significant advancements in the history of pyroptosis are summarized in Figure 1.

### 2.2 Activation of pyroptosis pathways

Pyroptosis has already been proven to play a crucial function in the immune response against various pathogens, including bacteria and viruses. Therefore, many substances, including inflammatory mediators, drugs, and so on, can activate pyroptosis. Detection of pathogen‐associated molecular patterns (PAMPs) and danger‐associated molecular patterns (DAMPs) by pattern recognition receptors (PRRs), initiates the typical process of pyroptosis.^3^, ^4^ In addition, immediately inflammatory substances like lipopolysaccharide (LPS) can trigger pyroptosis in a noncanonical inflammasome pathway. Extracellular LPS stimulates the expression of Type I interferon, which in turn activates the interferon Type I receptor and results in the synthesis of caspase 11.^18^, ^19^ Chemo‐drugs may activate caspase 3, triggering pyroptosis via the cleavage of GSDME^16^ Tumor necrosis factor‐alpha (TNF‐α) activates caspase 8 in tumor cells to activate pyroptosis.^20^ Granzyme, which functions as an inducer of pyroptosis, enters the target cells by way of perforin and is generated by cytotoxic T lymphocytes (CTLs) and natural killer (NK) cells.^21^

### 2.3 Key molecules and pathways involved in pyroptosis

Pyroptosis, an inflammatory mode of PCD, requires the protein families GSDM and caspase. The cleavage of GSDMs and the production of cytokines such as IL‐1β, IL‐18, and high mobility group box 1 (HMGB1), are the outcomes of pyroptosis.^5^, ^6^ In the end, this caused the cells to swell, break apart, and respond in an inflammatory cascade.

**FIGURE 2:**
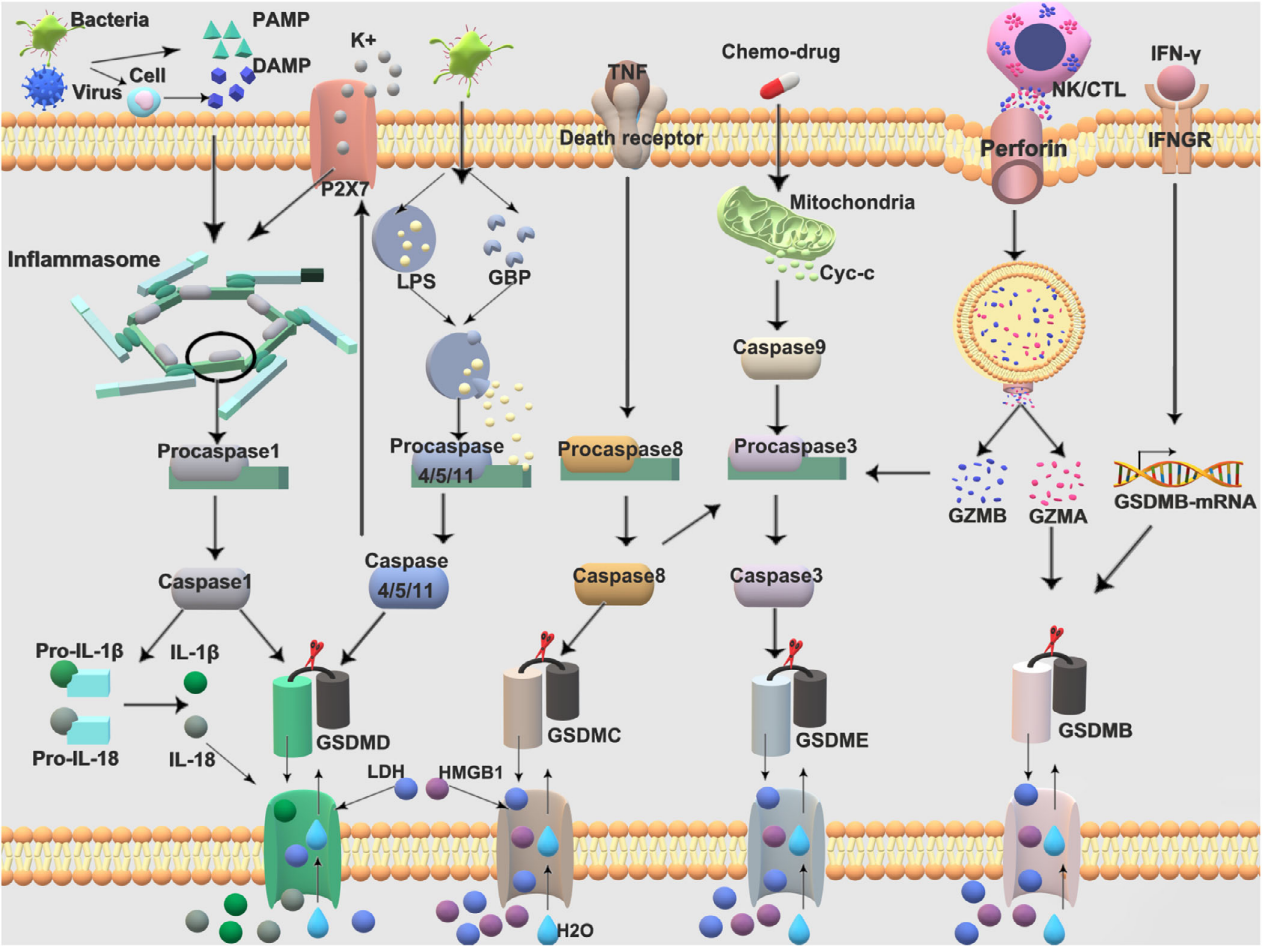
Schematic illustration of the different pyroptosis pathways. At present, there are mainly four distinct signaling pathways that have been identified to induce pyroptosis, including the classical caspase 1‐dependent pathway, which depend on the inflammasomes, nonclassical caspase 4/5/11‐dependent pathway, caspase 3/caspase 8‐mediated pathway, and granzymes‐mediated pathway. DAMP, danger‐associated molecular patterns; PAMP, pathogen‐associated molecular patterns; LPS, lipopolysaccharide; IL‐1β, interleukin‐1β; IL‐18, interleukin‐18; LDH, lactate dehydrogenase; HMGB1, high mobility group box 1; GZMA, granzyme A; GZMB, granzyme B; GBP, guanylate‐binding proteins; TNF, tumor necrosis factor; IFN‐γ, interferon‐γ; IFNGR, IFN‐gamma receptor; Cyc‐c, cytochrome c; NK, natural killer cells; CTL, cytotoxic T lymphocytes.

In addition to the early finding of the traditional caspase 1‐dependent pathway and the nonclassical caspase 4/5/11‐dependent pathway, recent studies illustrate more detail on newly discovered pathways that lead to pyroptosis, such as the caspase 3/caspase 8‐mediated pathway and granzymes‐mediated pathway (GZMA/GZMB). Figure 2 depicts the key molecules and pathways involved in pyroptosis. A thorough description of each pathway process for pyroptosis follows.

#### 2.3.1 Canonical pathway of pyroptosis

The canonical pathway is another name for the caspase 1‐dependent pathway. Pro‐caspase 1, the unreactive zymogen form of caspase 1, exists in the cytoplasm under normal circumstances. It is only activated when the canonical inflammasomes are functioning. Inflammasomes are multimolecular complexes composed of pro‐caspase 1, ASC, and PRRs.^22^ The majority of PRRs are NLRs, which include absent in melanoma 2 (AIM2), leucine‐rich repeat and pyrin domain‐containing protein 1 (NLRP1) and its murine homolog, NLRP1B, leucine‐rich repeat and pyrin domain‐containing protein 3 (NLRP3) and NLR family CARD domain‐containing protein 4 (NLRC4), and pyrin.^23^, ^24^

When PRRs detect DAMPs and PAMPs, the typical process of pyroptosis begins.^3^, ^4^ When tissue homeostasis is disrupted and monomeric PRR proteins oligomerize, PRRs undergo a conformational change to function as the oligomerization core.^25^ When activated, several PRRs containing caspase activation and recruitment domains (CARD), such as NLRC4.^26^ and NLRP1B ,^27^ may directly assemble pro‐caspase 1 via CARD–CARD interactions, removing the requirement for an adaptor molecule. Certain PRRs that do not include CARD must initially recruit ASC to recruit pro‐caspase 1. Due to its possession of both pyrin and CARD domains, ASC can act as a molecular bridge between proteins containing the pyrin domain (PYD), such as NLRP3, AIM2, and pyrin, and the CARD domain of pro‐caspase 1.^28^ Notably, NLRP1 can draw pro‐caspase 1 with or without the presence of ASC.^27^, ^29^ The structure and function of inflammasomes are discussed in further depth in Section 3. The proximity of the integral inflammasome increased caspase 1 activation via autoproteolysis during inflammasome assembly. According to recent research, the inflammasome–caspase 1 complex possesses an inherent self‐limiting mechanism that guarantees prompt caspase 1 inactivation.^30^ Caspases 1 transforms pro‐IL‐18 and pro‐IL‐1 into their mature forms.^31^ Moreover, caspase 1 cleaves GSDMD to produce an N‐terminal domain (GSDMD‐NT), which results in the formation of pores in the cell membrane.^6^ Influx of salt and water and subsequent cell swelling, is ultimately brought on by the pore. Meanwhile, it facilitates the release of mature IL‐1β, IL‐18, and HMGB1, all of which are inflammatory mediators. Recent studies have demonstrated that GSDMB is also involved in the pyroptotic canonical pathway in addition to GSDM. Caspase 1 cleaves GSDMB at position 236, leading to the generation of N‐terminal fragments, oligomerization, and pore formation in the cell membrane, eventually resulting in pyroptosis.^32^

#### 2.3.2 Noncanonical inflammasome pathway

In 2011, it was proven that infections with Gram‐negative bacteria cause pyroptosis through a novel noncanonical caspase 11 pathway.^19^ Compared with the conventional pathway, the crucial stage in the noncanonical inflammasome pathway is the activation of caspase 4/5/11, which is triggered immediately by inflammatory substances like LPS and does not require an inflammasome.^18^ Interferon (IFN) and LPS boost the expression of interferon‐inducible guanylate‐binding proteins (GBP) by activating the interferon receptor and Toll‐like receptor 4 (TLR4)/MD‐2.^33^ LPS may reach the cytoplasm due to the ruptured vacuoles induced by GBP.^34^ Human homologue caspase 4, human homologue caspase 5, and mouse caspase 11 display excellent specificity and affinity for binding to intracellular LPS. LPS and its lipid component A bind to caspase through the CARD module of the caspase.^35^ Caspase 4/5/11 activation provokes GSDMD and pyroptosis.^36^

In contrast to the canonical pathway, the noncanonical route cannot develop pro‐IL‐18 and pro‐IL‐1β directly.^19^ However, it may ultimately lead to the creation and emission of IL‐18 and IL‐1β by inadvertently activating caspase 1 and NLRP3. LPS triggers Pannexin‐I channel cleavage and caspase 11‐associated adenosine triphosphate (ATP) release, which sequentially activates ATP‐gated P2 × 7 and enhances K+ efflux as well as caspase 1/NLRP3 activation in macrophages.^37^ Caspase 11 decreases cellular potassium levels to activate NLRP3.^38^ Caspase 4 via potassium efflux will adequately activate the NLRP3 and speed up IL‐1β generation.^39^ GSDMB induces the noncanonical inflammasome pathway by directly interacting with the CARD domain of caspase 4 and gradually increasing caspase 4 activity. In the meantime, GSDMB overexpression promotes GSDMD cleavage, which is associated with an upsurge in lactate dehydrogenase (LDH) release.^40^

#### 2.3.3 Caspase 3/caspase 8‐mediated pathway

Apart from the caspase proteins mentioned above, pyroptosis can be triggered by other apoptotic caspases. Active caspase 3 cleaves the DFNA5/GSDME to generate a necrotic GSDME‐NT that punctures the plasma membrane and provokes pyroptosis after apoptosis is induced successfully, as described by Rogers et al. in 2017.^41^ According to the research conducted by Wang et al.^16^ in the same year, chemo‐drugs, including doxorubicin (DOX), cisplatin, and actinomycin‐D, may activate caspase 3, activating pyroptosis via the cleavage of GSDME. Although the caspase 3/GSDME route does not need the inflammasome, the synthesis of GSDME‐NT enables the NLRP3 inflammasome, which further activates the conventional pathway and boosts the growth of IL‐1β and IL‐18.^42^ Meanwhile, HMGB1 and LDH are released from pyroptotic cells by the GSDME.^43^, ^44^ GSDMB probably participates in cellular sulfatide transport because caspase 3/−6/−7 cleave GSDMB‐NT at 88DNVD91, which binds sulfatide‐containing liposomes.^45^

These findings contradict the traditional view that pyroptosis cannot be provoked only by inflammatory caspases. The activation of caspases, particularly caspase 3, was thought to be one of the essential characteristics of the apoptosis process in prior decades. We are currently aware that caspase 3 activation can occur without apoptosis. Caspase 3‐mediated pyroptosis and apoptosis share the same upstream signaling mechanism. This indicates that chemotherapy set off the transfer of BAX/BAK to the outer mitochondrial membrane, resulting in holes development and release of mitochondrial outer membrane permeabilization and cytochrome C.^46^ This activates caspase 9 and caspase 3 in turn.^47^ Simply put, GSDME expression levels are the primary determinants influencing the transition from pyroptosis to apoptosis. High levels of GSDME production cause GSDME to be cleaved into two terminals by active caspase 3, which appears in pyroptosis. Conversely, apoptosis will occur if GSDME expression is minimal.^48^, ^49^ Likewise, ATP induces pyroptosis by activating the caspase 3/GSDME pathway in macrophages when the classic NLRP3 pathway is blocked.^50^

TNF‐α activates caspase 8 in tumor cells. Caspase 8 can stimulate caspase 3. The metabolite‐ketoglutarate boosts the growth of the death receptor DR6, which is located in the plasma membrane and activates caspase 8. Pyroptosis rather than apoptosis occurs when caspase 8 cleaves GSDMC at D365 and releases its N‐terminal domain.^20^ Active caspase 8 cleaves both GSDMD and GSDME in Yersinia‐infected murine macrophages, ending in pyroptosis.^51^ In addition, caspase 8 cleaves GSDMC to create GSDMC‐NT, which triggers tumor necrosis by forcing cancer cells to undergo pyroptosis instead of apoptosis.^52^

#### 2.3.4 GZMA/GZMB

Granzymes play an important role in cytotoxic lymphocyte‐mediated immunity.^53^ Granzyme enters the target cells by way of perforin and is generated by CTLs and NK cells.^21^ They are considered to defeat cells via inducing apoptosis under prior studies.^54^ Granzymes have recently been revealed to also function as inducers of pyroptosis. According to research by Zhou et al.,^55^ GZMA from CTLs and NK cells destroys GSDMB to form membrane holes that trigger pyroptosis. IFN‐γ stimulates this process by raising GSDMB expression. This research identifies that GSDM‐mediated pyroptosis kills the cytotoxic lymphocyte, which may improve antitumor immunity. For GZMA‐mediated pyroptosis, the expression of GSDMB is necessary. GSDMB is expressed in both healthy tissues and malignant tissues.^56^ GZMB, which is generated by NK cells, evokes pyroptosis in tumor targets by cleaving GSDME at the same site as caspase 3. In addition, GZMB activates and cleaves caspase 3.^57^ The granzyme‐mediated pyroptosis pathway sheds fresh light on antitumor treatment.

### 2.4 Morphological changes in pyroptosis

The pathway described above ultimately leads to the cleavage of the GSDMs protein into its functional form, the N‐terminus domains. This enables it to fulfill its biological role. During pyroptosis, the N‐terminal domain translocates to the plasma membrane, where it can bind membrane lipids like phosphoinositide and cardiolipin, oligomerize in the membrane to make pores, and show cytotoxicity that breaks down the membrane.^15^, ^58^ Recent studies have shown that the pores formed in liposomes, with internal diameters of 10−20 nm, are large enough to allow the passing of IL‐1β, IL‐18, and other cellular proteins.^15^, ^59^ Lamkanfi and coworkers^60^ observe pyroptotic cell and create a model of pyroptotic cell disintegration. They demonstrate that early Ca2+ influx and mitochondrial degradation are conserved processes of GSDMD‐mediated pyroptotic cell death. These processes occur before the breakdown of the plasma membrane after activation of caspase 11. Ionic flow, cell swelling, mitochondrial depolarization, and lysosome leakage all take place during pyroptosis long before cell lysis with nuclear condensation.^60^ Eventually, the cell ruptures and water leaks inside, leading to cell swelling and rupture, the liberation of inflammatory chemicals inside the cell, and the induction of pyroptosis.

## 3 INFLAMMASOMES AND THEIR FUNCTIONS

Using the term “inflammasome,” Tschopp and coworkers^14^ defined a molecular complex present in activated immune cells' cytoplasm and contributing to the activation of the caspases 1. Inflammasomes are primarily utilized in inflammation and immunity. The inflammasome and the microenvironment of tumor cells are associated with the development of cancer. Thus, the regulation of inflammasome generation and activation is crucial for the pathogenesis, progression, and therapeutic response of cancer. In this section, we are going to give you a detailed review of how the inflammasome works and how it gets activated.

### 3.1 Structure of inflammasomes

**FIGURE 3:**
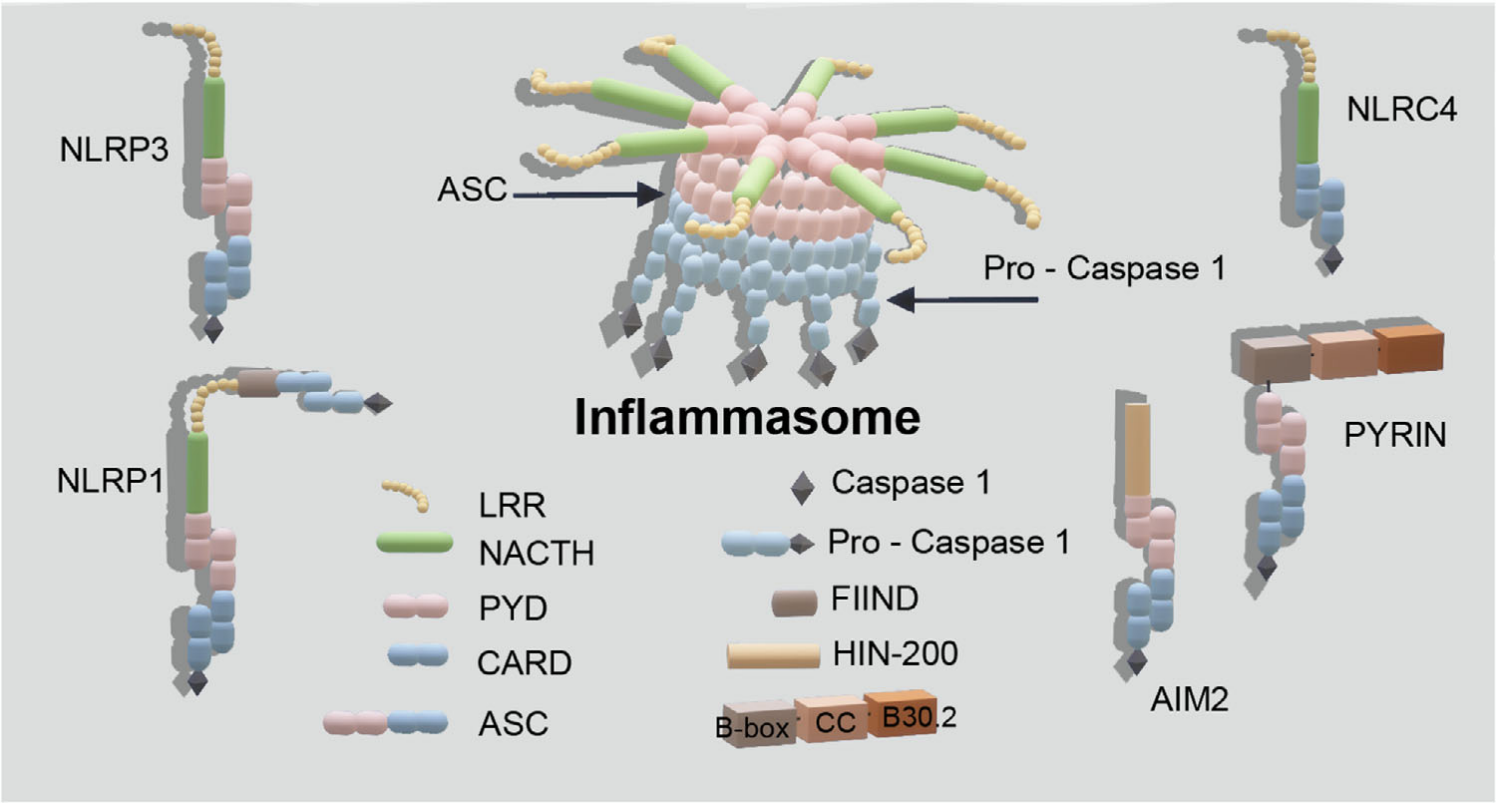
Structure of inflammasome. The structure of the five inflammasome (NLRP3, NLRP1, NLRC4, AIM2, and PYRIN). PYD, the pyrin domain; CARD, caspase activation and recruitment domains; LRR, leucine‐rich repeat; NACTH, central nucleotide‐binding and oligomerization domain; NLRP3, leucine‐rich repeat and pyrin domain‐containing protein 3; NLRP1, leucine‐rich repeat and pyrin domain‐containing protein 1; NLRC4, NLR family CARD domain‐containing protein 4; AIM2, absent in melanoma 2.

The inflammasome is a complex assembly that comprises PRRs, pro‐caspase 1, and ASC. NLRP3, NLRP1, NLRC4, AIM2, and PYRIN make up the majority of PRRs. Figure 3 depicts the basic structure of the inflammasome straightforwardly. NLRP3 is a member of the canonical inflammasomes that generate NLRs. An N‐terminal PYD, a C‐terminal leucine‐rich repeat (LRR), and a central nucleotide‐binding and oligomerization domain (NACTH) comprise NLRP3, but no C‐terminal CRAD.^61^ NLRP1 is unique among the members of this family. Besides the NACTH domain, N‐terminal PYD, and the C‐terminal LRR, NLRP1 encodes two more domains at its C terminus, a FIIND (function to find domain) and a CARD. The autolytic proteolytic cleavage of the C‐terminal FIIND structural domain is necessary for NLRP1 activity.^62^ A C‐terminal LRR domain, an N‐terminal CARD domain, and a NACTH domain encompass the NLRC4 protein. In addition to containing the PYD and CARD structural domains, the C‐terminal HIN‐200 domain of AIM2 serves as distinguishing features.^63^ PYRIN contains not only the PYD and card domains, but also a linker region connecting the B‐box, coiled‐coil (CC) and B30.2 domains. The oligomerization of pyrin is mediated by the CC and B‐box domains.^64^

### 3.2 Activation of inflammasomes and downstream signaling

There are a variety of substances that activate NLRP3, involving ATP, reactive oxygen species (ROS), potassium efflux, and internal sources of damage signal.^65^, ^66^ NACTH domain ATPase activity is necessary for the formation of NLRP3 oligomers following activation.^67^ The extensively used NLRP3 inhibitor MCC950 has recently been proven to target this ATPase activity. When the NLRP3 PYD attaches to the ASC PYD, the inflammasome is initiated.^28^ Inflammasome activation is mediated by the NLRP3 inflammasome sensor component in response to anomalies in membrane integrity, leading to pyroptosis along with the secretion of IL‐1β and IL‐18.^68^ Inflammasomes, especially NLRP3, are downregulated in dormant cells but upregulated by the NF‐κB pathway.^69^ Anthrax lethal toxin, muramyl dipeptide, and components of Toxoplasma gondii can activate NLRP1.^70^ NLRP1 activates pro‐caspase 1 in two ways. One of these pathways is that anthrax lethal factor cleaves NLRP1, which drives N‐terminal degradation and release of CARD at the C‐terminus as well as allowing NLRP1 to directly bind caspase 1 to create an inflammasome.^71^ Another pathway involves NACTH domain hydrolyses ATP, which changes the protein conformational of NLRP1, completes NLRP1 activation, and integrates it into the ASC‐dependent inflammasome. This pathway is activated when LRR binds double‐stranded DNA (dsRNA) from the virus.^72^ The Type III secretion system proteins and flagellin are recognized by NLRC4.^26^ AIM2 recognizes innate immune receptors on cell membranes as dsDNA generated in response to cellular disruption and pathogen assault. AIM2 triggers the inflammasome to mature and culminate in pyroptosis by detecting dsDNA. The C‐terminal HIN‐200 domain and the N‐terminal PYD of AIM2 are served as distinguishing features. While the HIN200 domain binds dsDNA from cellular damage and microbial infections, the PYD links with the ASC to cause pyroptosis.^63^ Pyrin serves to detect pathogen modification and Rho GTPase silencing.^73^ Pyrin has previously been demonstrated to contribute to the maturation of caspase 1 and the release of IL‐1β, which in turn causes pyroptosis.^74^ The PYRIN protein binds to the ASC via its N‐terminal PYD to fulfill its function.^64^

## 4 PYROPTOSIS AND INFLAMMASOMES IN CANCER

Pyroptosis and inflammasomes is closely linked to tumor immunity and the TME. Pyroptosis and inflammasomes is thus a promising entry point for research on targeted therapies. As research advances, inflammatory cell activities are becoming more well known and getting more attention in the TME. Pyroptosis has been identified as a double‐edged sword in studies on cancer etiology. Pyroptosis occasionally aids the malignant progression of cancer. Long‐term pyroptosis of cancer cells may occur from an adverse TME that accelerates the development of the disease. Chronic pyroptosis‐derived proinflammatory cytokines construct and maintain an inflammatory environment that encourages tumor growth.^75^ For instance, the early microenvironment of ovarian cancer may induce the production of DAMPs, which may subsequently activate AIM2 and NLRP3 inflammasomes. When triggered by microbial substances or endogenous cytokines, activation of NF‐κB causes overexpression of NLRP3, AIM2, pro‐IL‐1β, and pro‐IL‐18. Inflammasome activation, pyroptosis, and maturation of IL‐1β and IL‐18 all contribute to chronic inflammation in advanced stages.^76^ Pyroptosis, on the other hand, may postpone the development and progression of tumors. Acute and widespread pyroptotic activation results in a major invasion of immune cells that strengthens antitumor immunity to prevent tumor growth and trigger substantial cancer cell death.^77^

In this section, we will explore the impact of inflammasomes and mediators produced by pyroptosis on the TME and describe the application of pyroptosis in common tumors such as lung, breast, colorectal, gastric, and gynecological cancers. Other uncommon tumors will not be covered in this review due to space limitations.

### 4.1 Impact of inflammasomes on the TME

The NLRP3 inflammasome facilitates the invasion of myeloid cells into TMEs like myeloid‐derived suppressor cell (MDSC) and tumor‐associated macrophage (TAM), which benefits tumor progression.^7^ Inflammasome suppression elevated CD8+ T and CD4+ cell infiltration, decreasing TAM infiltration and amplifying the therapeutic effect of PD‐L1 inhibition in tumors with high levels of inflammasome signaling activity.^78^ An approach that targets NLRP3, specifically its ATPase activity, in the TME may offer new cancer treatment options. However, in subsequent chapters describing the application of pyroptosis in cancer, it was found that many drugs inhibit cancer by activating NLRP3 to initiate pyroptosis. The use of NLRP3 in cancer is currently controversial. Dipeptidyl peptidase inhibition activates the NLRP1 inflammasome, which triggers the secretion of proinflammatory cytokines and activates the response of the Th1 cell. The CXCL10/CXCR3 axis is further activated, which induces the infiltration of T and NK cells, strengthens the efficacy of anti‐PD1 antibodies, and inhibits tumor progression.^79^ In a subcutaneous mouse model of melanoma, NLRC4 is essential for the generation of cytokines in TAM as well as the development of IFN‐γ‐producing CD8+ and CD4+ T cells, both of which impede tumor progression.^80^ Innate immune detection of cytosolic DNA in DC via the STING pathway is one major mechanism activating spontaneous T cell responses against malignancies. The AIM2 inflammasome can inhibit chronic STING signaling by inducing pyroptotic cell death.^81^ AIM2 accelerated tumor development by reducing CD4+ effector T cells while increasing the number of Treg cells. Moreover, IL‐1 and IL‐18 production needed AIM2, which enhanced Treg accumulation and tumor progression in vivo.^82^ A linker region domain of PYRIN has been demonstrated to interact with the proline serine threonine phosphatase‐interacting protein (PSTPIP1/CD2BP1), which is crucial for the structure of the cytoskeleton.^83^

### 4.2 Influence of pyroptosis on the TME

When pyroptosis is activated, inflammatory mediators, such as IL‐1β, IL‐18, and HMGB1, are produced, all of which can have a positive impact on the progression of the cancer.^84^, ^85^, ^86^ Yet, several investigations have indicated that IL‐1β, IL‐18, and HMGB1 have a suppressive impact on cancer.^85^, ^87^, ^88^ These conflicting findings indicate that the impact of pyroptosis on the TME is complicated and context dependent. To better understand the role of pyroptosis in the TME and its potential as a therapeutic target for cancer treatment, more research is needed. The next section explores how pyroptosis affects the TME.

#### 4.2.1 IL‐1β

Inflammatory signals activate IL‐1β in a variety of immune cells. In the TME, IL‐1β has dual functions that are connected to the growth of tumors. IL‐1β signaling activates innate immune cells as well as antigen‐presenting cells by polarizing CD4+ T cells toward Th1 and Th17 cells and activating them.^89^, ^90^ IL‐1β has been attributed to have a significant benefit in terms of treating acute inflammations and evoking specific anticancer defenses. For instance, NLRP1 (−/−) mice exhibit increased inflammation and cancer burden when IL‐1β levels are low.^91^ TAMs and MDSCs were enhanced in the immunosuppressive environment induced by IL‐1β, which promotes tumor growth.^85^ Dendritic cells (DCs) can be differentiated from monocytes, and IL‐1β stimulates their expansion. Besides, IL‐1β provokes hyperactive DCs to foster tumor lysates as immunogens and attach them to lymphocytes to trigger cytotoxic CD8+T cell responses peculiar to the antigen.^92^, ^93^

#### 4.2.2 IL‐18

Both procancer and anticancer properties are discovered in systemic, multifunctional cytokine IL‐18. It is initially recognized as a potent IFN‐production promoter.^84^ It enhances the Th1 immune response and activates T and NK cells to generate IFN‐γ, which potentially assists with tumor immunity. Th1 differentiation is facilitated by this cytokine in conjunction with IL‐12.^94^ IL‐18 can support angiogenesis, metastasis, and growth. Moreover, it is capable of evading the immune system.^84^ When an immune‐suppressing microenvironment is established, IL‐18 boosts tumor growth even further. According to Lim et al.,^95^ IL‐18 dramatically enhances monocytic MDSC (M‐MDSC) through CD11b (−) bone marrow progenitor cell differentiation, suppressing in vitro T cell expansion and IFN production. IL‐18, which is mediated by the NLRP3 inflammasome, elevates NK cell maturation, surface expression of the death ligand FasL, and the ability to eradicate FasL‐sensitive malignancies.^96^

#### 4.2.3 HMGB1

Pyroptosis generates HMGB1, a variable nuclear protein that plays a part in DNA recombination repair, transcription, and chromatin remodeling.^97^ Extracellular HMGB1 activates the NLRP3 inflammasome after it interacts with several sensors and begins NF‐κB signaling.^98^, ^99^ Besides, HMGB1 signaling via TLR2 and TLR4 receptors released cytokines, including IL‐6, TNF, and IL‐8, that are necessary for CD8 + T cell activation.^100^ HMGB1 seems to have both tumor‐promoting and tumor‐suppressive properties in malignancies. Neo‐angiogenesis and cancer immunity are both increased. HMGB1, a substance generated by dying tumor cells, interacts with TLR4, a receptor crucial for both innate and adaptive immune responses, to initiate the processing of tumor antigens by mature DCs and the anticancer immune response.^101^ At the same time, the immunosuppressive TME aids in the survival of tumor cells with HMGB1 by enhancing the viability and recruitment of MDSCs.^86^, ^102^

#### 4.2.4 GSDMs proteins

In response to diverse stimuli, GSDMs proteins rupture cell membranes, triggering the release of proinflammatory cytokines. This eventually leads to pyroptotic cell death. Immune signaling and cytokines influence GSDMs. For example, IFNα, IFNβ, IFNγ, and TNFα elevate GSDMB expression and stimulate pyroptosis.^55^ IRF2, a transcription factor from the interferon regulatory factor (IRF) family, binds the GSDMD promoter and induces the expression of GSDMD and pyroptosis.^103^

GSDME suppresses tumors by inducing pyroptosis, which produces IL‐18, IL‐1β, and HMGB1 to convert “cold” tumors into “hot” tumors, promoting antitumor immunity. In mice with GSDME‐expressing tumors, knocking out GSDME increases tumor development, whereas ectopic expression inhibits tumor development. This tumor suppression is mediated by killer cytotoxic lymphocytes, which are deficient in perforin‐deficient or lymphocyte‐depleted mice. The expression of GSDME leads to NK cells and CD8^+^ T cells infiltrate the tumor more frequently and functioning better, as well as TAMs phagocytose more effectively.^57^ Chimeric antigen receptor (CAR) T cells deliver granzyme, which cleaves GSDME and activates caspase 3 in target cells. Similarly, pyroptosis elements trigger macrophages to activate caspase 1 for GSDMD cleavage, which causes the release of cytokines and the accompanying cytokine release syndrome (CRS). The amount of perforin/enzyme B used by CAR T cells, but not by preexisting CD8(+) T cells, is particularly critical for CAR T cells to induce pyroptosis in the target cell.^104^ Using tumor cells expressing high levels of GSDME from lung, liver, breast, and glioma cancers, Cao et al.^105^ showed that irradiation caused pyroptosis through caspase 9/caspase 3/GSDME signaling. The combination of chemotherapeutic drugs and irradiation increased the incidence of pyroptosis substantially. GSDME‐overexpressing tumors had heightened antitumor immunity, as evidenced by noticeably higher quantities of CTLs and the secretion of related cytokines.^105^ We summarized the role of inflammasomes and pyroptosis‐related proteins in regulating immune cells as described previously in Table 1.

**TABLE 1:** The role of inflammasomes and pyroptosis‐related proteins in in immunity.

Inflammasomes or pyroptosis‐related proteins	Role in immunity	References
NLRP3	Invasion of TAMs, MDSCs↑ Suppressant elevated CD8+ and CD4+ T cells infiltration	^7^, ^78^
NLRP1	Response of Th1 cells ↑ Infiltration of T and NK cells ↑	^79^
NLRC4	Essential for CD8+ T cells, CD4+ T cells and TAMs	^80^
AIM2	Treg↑ CD4+ effector T cells↓	^82^
IL‐1β	Th1 cells, Th17 cells↑, TAMs↑, MDSC↑ DC↑	^89^, ^90^ ^85^ ^92^, ^93^
IL‐18	Immune response↑ : Th1 cells response↑, IFN‐γ↑ Immune response↓: M‐MDSCs↑, IFN‐γ↓	^94^ ^95^
HMGB1	Trigger dendritic cell tumor antigen processing *MDSCs↑	^101^ ^102^
GSDME	NK cells, CD8+ T cells and TAMs ↑ *cytotoxic T lymphocytes	^57^ ^105^

### 4.3 Pyroptosis in lung cancer

Globally, lung cancer accounts for 18.4% of all cancer deaths, causing enormous societal burdens and economic losses.^8^ In lung cancer, the avoidance of apoptosis or the induction of other PCDs has been a focus of research and is essential for the discovery of innovative treatment strategies. P53‐induced pyroptosis is a viable therapeutic strategy for the treatment of non‐small cell lung cancer (NSCLC).^106^ GSDMD protein levels are dramatically increased in NSCLC, which is correlated with aggressive traits like larger tumor sizes and advanced TNM stages.^107^ In CTLs, the expression of GSDMD has a favorable association with CD8^+^T cell markers. GSDMD colocalizes with GzmB near immune synapses to ensure the best CTL response to lung cancer cells.^108^ In lung adenocarcinoma (LUAD), a high expression of GSDMC is an indication of a poor prognosis.^109^ In NSCLC immunotherapy, the chimeric costimulatory converting receptor‐modified NK92 cell (CCCR‐NK92) is capable of converting the negative PD1 signal into an activating signal, leading to GSDME‐induced pyroptosis.^110^

Pyroptosis has been discovered to contribute to the resistance of lung cancer chemotherapy. It may be possible to overcome treatment resistance and improve patient outcomes by understanding the relationship between pyroptosis and lung cancer chemotherapy. Peng et al.^111^ prove that the knockout of GSDME induces a transition from apoptosis to pyroptosis. GSDME expression is reduced, whereas overexpression increases chemosensitivity in lung cancer tissue. Furthermore, higher GSDME expression facilitates chemokine release, which has an impact on T‐cell activation and infiltration.^111^ Both paclitaxel and cisplatin induce pyroptosis in lung cancer cells, with cisplatin causing more pyroptosis. In addition, cisplatin may provide significant benefits in the treatment of lung cancers with high GSDME expression.^49^ These findings emphasize the potential immunotherapeutic implications of targeting GSDME in the treatment of lung cancer to minimize chemotherapy resistance.

Besides, pyroptosis in lung cancer can be induced or suppressed by many drugs, noncoding RNAs, and inhibitors. Cucurbitacin B (CuB), a compound extracted from muskmelon pedicel, inhibits tumor growth in NSCLC cells by directly binding to TLR4, activating the NLRP3 inflammasome and inducing pyroptosis.^112^ Polyphyllin VI (PPVI), an active saponin, suppresses NSCLC proliferation by triggering caspase 1‐dependent pyroptosis via the ROS/NF‐κB/NLRP3/GSDMD signaling axis.^113^ Simvastatin, an antihyperlipidemic drug, triggers pyroptosis to inhibit proliferation and migration in NSCLC.^114^ In lung cancer cells, dasatinib initiates pyroptosis and p53‐independent elevations in GSDMD and GSDME protein levels.^115^ As reported by Lu et al.,^116^ GSDME overexpression made targeted small molecule inhibitors more sensitive. The widespread cooccurrence of apoptosis and pyroptosis in lung cancer cells is highlighted by factors such as Kirsten rat sarcoma virus, epidermal growth factor receptor, or anaplastic lymphoma kinase, stimulating apoptosis and pyroptosis in NSCLC cells.^116^ In human NSCLC, LncRNA‐XIST is found to be upregulated, particularly in cisplatin‐treated tumors. XIST promotes cell viability and raises DDP chemoresistance by preventing SMAD2 from moving to the nucleus, inhibiting p53 and NLRP3 transcription.^117^ While knocking down XIST promotes pyroptotic cell death in NSCLC through miR‐335/SOD2/ROS signaling.^118^ Downregulation of NLRP3 may prohibit the pyroptotic cell death caused by the knockdown of miR‐556‐5p.^119^

As the investigation progressed, Xie et al., the researchers applied pyroptosis to nanomedicine in the treatment of lung cancer, created inhaled poly (lactic‐co‐glycolic acid) porous microspheres loaded with decitabine (DAC) and DOX (denoted as CO‐MPs) to induce lung cancer pyroptotic cell death with fewer systemic side effects. In mechanism, CO‐MPs promoted pyroptosis by reversing GSDME silencing and upregulating caspase 3 expression. Consequently, CO‐MPs suppress lung tumor growth and promote immunological memory.^120^

The application of pyroptosis in the therapy of lung cancer is so promising that clinical trials have already been carried out. Several late‐stage anticancer clinical trials are investigating. RRx‐001 (generic name: bromonitrozidine) has been confirmed safe and well tolerated in humans, along with potential effectiveness in various diseases resulting from immune and inflammasome activation.^121^ The Phase 2 trial of RRx‐001 showed that it not only made platinum‐resistant SCLC sensitive to chemotherapy, but also reduced the side effects of platinum.^122^ In an ongoing Phase 3 trial, RRx‐001 is being tested in combination with carboplatin/cisplatin and etoposide for the treatment of SCLC.^123^

Pyroptosis‐related genes have potential therapeutic applications, but they are also beneficial in the prediction of lung cancer prognosis. GSDME, caspase 3, and caspase 8 are more abundantly expressed in tumor tissue. Elevated GSDME expression is associated with a higher postoperative survival rate and a lower lymph node metastasis rate, which suggests that it may serve as a predictor of prognosis in lung cancer patients.^124^ LUAD prognosis is also associated with new pyroptosis‐related prognostic markers, including NOD1, NLRP1, NLRP2, NLRP7, and caspase 6. The notion that pyroptosis can mediate the TME to exert an antitumor effect is supported by these genes, which are intimately linked to immune infiltration.^125^ We have summarized the mechanisms and effects of pyroptosis‐related targets/agents in lung cancer in Table 2.

**TABLE 2:** Mechanisms and effects of pyroptosis‐related target/agents in cancer.

Cancer	Target/agents	Type	Signal and molecules	pyroptosis	References
Lung cancer	CuB	Extracts	TLR4, NLRP3	↑	^112^
	PPVI	An active saponin	ROS/NF‐κB/NLRP3/GSDMD/caspase 1	↑	^113^
	Simvastatin	Marketed medicine	∖	↑	^114^
	LncRNA‐XIST	Noncoding RNAs	p53 and NLRP3↓ miR‐335/SOD2/ROS	↓	^117^ ^118^
	miR‐556‐5p	Noncoding RNAs	Nlrp3↑	↑	^119^
	Dasatinib	Targeted drug	GSDMD GSDME↑	↑	^115^
	CO‐MP	Nano drug	GSDME↑ caspase 3↑	↑	^120^
	RRX‐001	Medicine in trial	inflammasome activation		^121^
Breast cancer	Cisplatin	chemotherapeutic agent	lncRNA MEG3/NLRP3/caspase 1/GSDMD		^128^
	Decitabine	Chemotherapeutic agent	GSDME↑	↑	^129^
	Doxorubicin	Chemotherapeutic agent	ROS↑ Caspase 3/GSDME	↑	^130^
	metformin	Marketed medicine	AMPK/SIRT1 caspase 3/GSDME	↑	^131^
	DHA	Marketed medicine	AIM2/caspase 3/DFNA5 caspase 1/GSDMD	↑	^132^
	Nobiletin	RORs agonist	miR‐200b/JAZF1/NF‐κB	↑	^135^
	Polydatin	Extracts	JAK2/STAT3↓ NLRP3, caspase 1, IL‐1β, IL‐18	↑	^137^
	As4O6	Oxide	ROS↑, P‐STAT3↓ caspase 3/GSDME	↑	^138^
	RIG‐1 agonist	Agonist of RIG‐1	STAT1, NF‐κB	↑	^139^
	BNP (ICG/DAC)	Nanomedicine	Caspase 3/GSDME	↑	^143^
	MPNPs (oncolytic viruses)	Nanomedicine	GSDME	↑	^144^
Gastric cancer	Famotidine	Marketed medicine	NLPR3, IL‐18, GSDME	↑	^159^
	Icariin	Medicine in trial	hsa_circ_0003159/miR‐223‐3p/NLRP3 axis	↑	^160^
	Simvastatin	Marketed medicine	Caspase 3/GSDME	↑	^161^
	Diosbulbin‐B	Extracts	Inhibit PD‐L1 and activate NLRP3	↑	^163^
	BIX‐01294	Inhibitor of histone methyltransferase	GSDME	↑	^164^
	LncRNA ADAMTS9‐AS2	Noncoding RNAs	miR‐223‐3P↓/NLRP3↑	↑	^165^
	LP‐R/C@AC	Nanomedicine	VEGFR2/STAT3	↑	^166^
	ZIF‐8@TPZ/Ce6	Nanomedicine	ROS↑	↑	^167^
Colorectal cancer	lobaplatin	Chemotherapeutic agent	Caspase 3, GSDME	↑	^177^
	DAC	Chemotherapeutic agent	Inflammasome↑	↑	^178^
	Secoisolariciresinol diglucoside	Extracts	Caspase 1, GSDMD,	↑	^179^
	Arsenic trioxide & ascorbic acid	Marketed medicine	ROS↑, caspase 1	↑	^180^
	GW4064	Agonist of an FXR	BAX/caspase 3/GSDME	↑	^181^
	T22‐PE24‐H6	Nanomedicine	GSDMD	↑	^182^
	DAC‐nano drugs/LipoDDP	Nanomedicine	caspase 3/GSDME	↑	^184^
Endometrial Cancer	HKDC1	Proteins	ROS↑ HOXC‐AS2/miR‐876‐5p/HKDC1	↑	^189^
	hydrogen	GAS	ROS↑ pyroptosis‐related protein↑	↑	^191^
Cervical cancer	SIRT1	Proteins	AIM2↓	↓	^196^
	Lobaplatin	chemotherapeutic agent	Caspase 3/GSDME	↑	^198^
	Δ‐Ru1 & Taxol	Nanomedicine	Caspase 1/GSDMD	↑	^199^
	Tanshinone II A	Targeted drug	miR‐214/GSDMD	↑	^201^
	miR‐214	Noncoding RNAs	NLRP3↑	↑	^202^
Ovarian cancer	LncRNA GAS5	Noncoding RNAs	ASC, caspase 1	↑	^218^
	lncRNA HOTTIP	Noncoding RNAs	NLRP1 、	↓	^217^
	LncRNA DICER1‐AS1	Noncoding RNAs	Pyroptosis‐related	Not investigated	^219^
	FOSL2	Gene	ASC, caspase 1, IL‐1β, IL‐18	↓	^220^
	BI 2536	Medicine in trial	Caspase 3/GSDME	↑	^43^
	Bexarotene	Marketed medicine	Caspase 4/GSDME	↑	^222^
	Nobiletin	RORs agonist	GSDMD/GSDME, IL‐1β, ASC	↑	^223^
	Osthole	Inhibitor of histamine H1 receptor	GSDME	↑	^225^
	α‐NETA	Inhibitor of choline acetyltransferase	GSDMD/caspase 4.	↑	^227^
	Citric acid	Marketed medicine	TXNIP, caspase 4, NLRP3, GSDMD	↑	^228^

### 4.4 Pyroptosis in breast cancer

Breast cancer causes significant harm to women's health, ranking second among cancer‐related deaths in women.^8^ A new treatment strategy for patients with breast cancer is offered by research on pyroptosis in the breast. As in other cancers, GSDME is substantially downregulated in breast cancer and inhibits cell proliferation and invasion. Furthermore, GSDME methylation is only identified in cell lines that express the estrogen receptor.^126^ Breast cancer cell proliferation and colony formation are negatively impacted by CDK7 inhibition in a p53‐GSDME‐dependent manner.^127^

Several investigations have found that different chemotherapy drugs can stimulate distinct pyroptosis pathways. Cisplatin enhances complete response rates in patients with triple‐negative breast cancer patients by activating the lncRNA MEG3/NLRP3/caspase 1/GSDMD pyroptosis pathway.^128^ Decitabine triggers GSDME expression by DNA demethylation, boosting pyroptosis and the chemosensitivity of MCF‐7/Taxol cells to Taxol.^129^ TNFα‐induced apoptosis is converted to pyroptosis by antibiotic chemotherapy drugs (daunorubicin, DOX, epirubicin, and actinomycin D) that activate PD‐L1. The interaction between p‐Stat3 and PD‐L1 during hypoxia facilitates its nuclear translocation, increasing GSDMC expression and triggering the GSDMC/caspase 8 pyroptosis pathway.^52^ DOX treatments elevate ROS accumulation, which activates the phosphorylation of JNK and causes pyroptosis via the caspase 3/GSDME pathway.^130^

Along with chemotherapy drugs, other drugs used in the treatment of breast cancer are related to pyroptosis. Metformin treatment greatly decreased breast cancer cell, colon cancer cell, and liver cancer cell viability and induced pyroptosis by improving the AMPK/SIRT1 pathway, upregulating NF‐κB p65 expression, and cleaving GSDME with caspase 3,^131^ Docosahexaenoic acid (DHA) may trigger pyroptosis by boosting NF‐κB nuclear translocation, caspase 1, and GSDMD activation,^132^ as well as activating the AIM2/caspase 3/DFNA5 pathway,^133^ Nobiletin (NOB), a retinoid acid receptor‐related orphan receptors (RORs) agonist,^134^ induces the pyroptosis of breast cancer cells via the miR‐200b/JAZF1/NF‐κB axis to stop tumorigenesis.^135^ Polydatin, a monocrystalline compound isolated from Polygonum cuspidatum Sieb,^136^ blocks the JAK2/STAT3 pathway and raises the expression of NLRP3, caspase 1, IL‐1β, and IL‐18 to contribute to the activation of pyroptosis in triple‐negative breast cancer.^137^ Tetraarsenic hexoxide (As4O6) could increase mitochondrial ROS generation by preventing STAT3 phosphorylation, causing caspase 3/GSDME‐dependent pyroptotic cell death and ultimately inhibiting tumor proliferation and metastasis in triple‐negative breast cancer cells.^138^ In breast cancer, retinoic acid‐inducible gene I (RIG‐I) agonist therapy revealed a significant immunogenic and therapeutic effect. RIG‐1 stimulated pyroptosis, increased lymphocytes, and decreased tumor growth via triggering STAT1 and NF‐κB.^139^ PolyI: C, a frequently employed synthetic dsRNA analogue that activates RIG‐I, facilitated triple‐negative breast cancer cell death by inhibiting the antipyroptotic function of TGF‐β.^140^ Many nanomaterials in combination with PolyI: C can lead to tumor cell death. However, the mechanism of pyroptosis has not been investigated.^141^, ^142^

Nanomedicine was employed to treat breast cancer with pyroptosis. Zhao et al. created a biomimetic nanoparticle (BNP) that contained indocyanine green (ICG) and DAC. ICG‐activated caspase 3 and DAC increased GSDME expression by inhibiting DNA methylation Through low‐dose photo‐activation. In the end, this photo‐activated pyroptosis of nanodrug generates an impressive systemic antitumor immunity for tumor inhibition.^143^ Su et al.^144^ utilized oncolytic viruses in conjunction with inhibitor nano prodrugs MPNPs to promote GSDME‐mediated pyroptosis, reversing an immunosuppressive TME and augmenting the effectiveness of anti‐PD‐1 treatment. Following a study, anti‐GSDMB antibody loaded into hyaluronic acid biocompatible nanocapsules has a significant and targeted impact on HER2‐positive cancers that overexpress GSDMB. These effects include decreased migration, increased sensitivity to trastuzumab, reduced tumor growth, and lowered lung metastasis by elevating GSDMB binding to sulfatides.^145^

Pyroptosis‐related genes can be exploited as prognostic predictors and therapeutic targets for breast cancer. In the study of prognostic‐related gene expression, breast cancers showed considerably lower levels of the pyroptosis pathway effector proteins caspase 1, IL‐1β, and GSDMD compared with nearby normal tissue.^146^ A study has found that GSDMB is a significant predictor of poor prognosis and therapeutic response in HER2‐positive breast cancer. GSDMB expression was linked to a poor prognosis and treatment response in HER2‐positive breast cancer. Additionally, GSDMB expression promoted cell viability and resistance to trastuzumab therapy.^147^ GSDME methylation is a promising biomarker for the diagnosis and prognosis of breast cancer.^148^ The prognosis of breast cancer can be independently attributed to three‐gene regression models involving IL18, GSDMC, and TIRAP. In contrast, a high‐risk score is associated with poorer progression, overall survival and relapse rates, while a low‐risk score is associated with immune cell infiltrations and immune checkpoints. Table 2 provides a summary of the mechanisms and effects of pyroptosis‐related targets and agents in breast cancer.

Immune checkpoint inhibitors may be better for use in combination with chemotherapy drugs than they are for use alone.^149^, ^150^ We hypothesized that it is the immune checkpoint inhibitors that enhance the induction of pyroptosis by chemotherapeutic agents, as pyroptosis is immunologically related. Therefore, the molecular and immunological mechanisms of chemotherapy‐induced pyroptosis need to be investigated in depth.

### 4.5 Pyroptosis in gastric cancer

Gastric cancer is the fifth most common cancer in the globe, with more than 1.08 million new cases diagnosed annually. It is also the fourth greatest cause of cancer‐related fatalities worldwide, accounting for 770 thousand deaths each year.^151^ The search for innovative and effective treatments for gastric cancer is particularly critical. GSDMA, GSDMC, and GSDMD are down‐regulated in gastric cancer,^152^, ^153^ although GSDMB levels are high.^154^ Reduced GSDMD expression speeds up tumor proliferation and S/G2 transition of cells by stimulating the STAT3 and PI3K/PKB pathways.^153^ GSDME is essential to the treatment of gastric cancer, as it is in other cancers. Wang et al.^155^ indicated that GSDME converted caspase 3‐dependent apoptosis into pyroptosis caused by chemotherapeutic agents 5‐FU. Intracellular mucin 20 variant 2 (MUC20v2) expression maintains mitochondrial calcium homeostasis and mitochondrial membrane potential (MMP), promoting cell viability and chemotherapeutic resistance by suppressing pyroptosis and apoptosis in gastric cancer cells.^156^ Pyroptosis and inflammation are inextricably linked. Infection with Helicobacter pylori infection is a major factor in gastric cancer. CagA, a Helicobacter pylori virulence factor, may trigger the migration and invasion of gastric cancer cells by activating the NLRP3 inflammasome.^157^ Li et al.^158^ have reported that the antibacterial activity of Callicarpa nudiflora can protect the gastric epithelium from Helicobacter pylori by inhibiting the ROS/NLRP3/caspase 1/IL‐1β signaling axis. Existing studies have shown that pyroptosis may both promote and inhibit gastric cancer, therefore more research into the connection between gastric cancer and pyroptosis is required.

Pyroptosis has been implicated in gastric cancer. Thus, numerous drugs and noncoding RNAs have been studied for their function in gastric cancer pyroptosis. Famotidine, for instance, stimulated cell pyroptosis in gastric cancer cells by activating NLPR3 inflammasomes, which resulted in increased IL‐18 release and GSDME expression.^159^ Icariin, the primary active ingredient of Epimedium, controlled the hsa_circ_0003159/miR‐223‐3p/NLRP3 axis to suppress gastric cancer and trigger pyroptosis.^160^ As it did in lung cancer, simvastatin caused pyroptotic cell death in gastric cancer by upregulating the expression of caspase 3 and GSDME.^161^ Low‐dose Diosbulbin‐B, a diterpene lactone isolated from *Dioscorea bulbifera* L,^162^ boosted the sensitivity of gastric cancer cells to cisplatin via inhibiting PD‐L1 and activating NLRP3‐mediated pyroptosis.^163^ When combined with cis‐platinum, BIX‐01294 (BIX), a specific inhibitor of euchromatic histone‐lysine N‐methyltransferase 2 (EHMT2) histone methyltransferase, lowers cell viability in gastric cancer cells through GSDME‐mediated pyroptotic cell death.^164^ LncRNA ADAMTS9‐AS2 suppresses gastric cancer cells and raises cisplatin sensitivity by inhibiting miR‐223‐3p, which in turn stimulates NLRP3 expression and eventually enhances pyroptotic cell death.^165^

Nanocomplexes have been widely explored in the pyroptosis of gastric cancer. Long et al.^166^ created a pH‐responsive liposome (Liposome‐PEO, LP), which contained apatinib and cinobufagin, and was covered with a hybrid membrane (R/C). This nanocomplex **LP‐**R/C@AC successfully cured gastric cancer, improved drug effects, reduced side effects, and had superior solubility and targeting ability. It mechanically inhibited tumor growth through the VEGFR2/STAT3 pathway and stimulation of pyroptosis.^166^ During the past few years, sonodynamic therapy has become a popular treatment in cancer research due to its noninvasive nature. Yu et al.^167^ have established a nanodrug that integrates zeolitic imidazole frameworks‐8 (ZIF‐8), encapsulated Chlorin e6 (Ce6), and tirapazamine (TPZ) to provide synergistic sonodynamic chemotherapy. It was also changed by the cytomembrane of gastric cancer cells for targeting capabilities. With ultrasound irradiation, this complex (ZIF‐8@TPZ/Ce6 (ZTC)) might generate ROS, induce pyroptosis, and perform antitumor functions.^167^

Wang et al.^168^ created a predictive signature related to pyroptosis. They discovered four novel pyroptosis‐related lncRNAs, namely HAND2‐AS1, LINC01354, RP11‐276H19.1, and PGM5‐AS1, with high expression correlated with a poor prognosis.^168^ In a different investigation, Liang et al. looked into PRGs in gastric cancer and developed a ten‐gene prognostic model (BIRC2, CD274, IRGM, ANXA2, GBP5, TXNIP, POP1, GBP1, DHX9, and TLR2). The study suggests that pyroptosis‐related risk signals can predict gastric cancer prognosis and identify immune cell infiltration in high‐risk patient tissues.^169^ The mechanisms and effects of pyroptosis‐related targets and substances in gastric cancer are summarized in Table 2.

### 4.6 Pyroptosis in colorectal cancer

Colorectal cancer is the third most prevalent cancer and the fourth main cause of cancer death.^170^ Inflammation is intimately connected to cancer, particularly colon cancer, and patients suffering from ulcerative colitis have a considerably elevated risk of colorectal cancer.^171^ According to Allen et al.,^172^ components of the inflammasome are protective against both recurrent and acute colitis as well as colitis‐associated cancer (CAC). According to bone marrow reconstitution experiments that have been performed, the NLRP3 gene, which is expressed in hematopoietic cells rather than intestinal epithelial or stromal cells, may be important for tumorigenesis prevention.^172^ Microbiome‐derived stimuli can enhance the cleavage of GSDMs into an active state, inducing pyroptosis and facilitating the release of inflammatory mediators. Eventually, the tumor burden is reduced, or a tumor‐promoting microenvironment is established. Moreover, GSDMB can generate pores in bacterial membranes, triggering intracellular pathogens to die.^173^ In different research, GSDME cleavage promoted pyroptosis and inhibited HCT116 cell viability by apoptin, a protein encoded by the chicken anemia virus VP3 gene.^174^ In colorectal cancer, GSDMD levels are decreased, and these levels are negatively correlated with tumor metastasis, tumor aggressiveness, and 5‐year survival rates.^175^, ^176^ Yet other articles suggest that pyroptosis contributes to colon cancer. GSDME‐mediated pyroptosis accelerates the progression of CAC by releasing HMGB1, which stimulates tumor cell proliferation and PCNA expression via the ERK1/2 pathway.^44^

Surprisingly, many chemotherapeutic medications and other agents suppress cancer through pyroptotic mechanisms. In a dose‐dependent manner, lobaplatin‐induced caspase 3‐GSDME‐dependent pyroptosis lowers the viability of colorectal cancer cells.^177^ DAC treatment can inhibit colon cancer growth by stimulating inflammasome expression and causing pyroptosis.^178^ The enhancement of the GSDMD‐dependent pyroptosis brought on by LPS increases the chemosensitivity of oxaliplatin in colon cancer cells.^176^ Secoisolariciresinol diglucoside, the main lignan in wholegrain flaxseed, reduced cell viability and triggered pyroptosis by improving cleavage of the N‐terminal fragment of GSDMD and promoting caspase 1 cleavage, which is connected with the ROS/PI3K/AKT/BAX mitochondrial apoptotic pathway.^179^ The combination of arsenic trioxide and ascorbic acid substantially inhibits colorectal cancer cell viability by producing ROS, upregulating caspase 1 expression, and promoting the development of inflammasomes to induce pyroptosis.^180^ GW4064, a Farnesoid X receptor (FXR) agonist, increased the chemosensitivity of cells to oxaliplatin by activating BAX/caspase 3/GSDME‐mediated pyroptosis.^181^

A nanoparticle‐based approach known as T22‐PE24‐H6 and T22‐DITOX‐H6 was created by the researchers. It targets colorectal cancer cells, inhibits tumor growth, and blocks lymphatic and hematogenous metastasis by triggering pyroptotic activation.^182^, ^183^ Zhang and coworkers^184^ combine DAC with chemotherapeutic nano drugs to predemethylate and upregulate the GSDME in colon adenocarcinoma cells. After that, by delivering cisplatin‐loaded nanoliposomes (LipoDDP) to tumor cells, drugs are delivered to activate the caspase 3 pathway and induce pyroptosis to achieve excellent tumor suppression. Table 2 provides a summary of the mechanisms and effects of pyroptosis‐related targets and agents in colorectal cancer.

In conclusion, these investigations shed light on the presumed mechanisms and function of pyroptosis in colorectal cancer. Consequently, these findings may offer an ideal method for improving the treatment and prognosis of this malignancy.

### 4.7 Pyroptosis in gynecological oncology

The three most common types of gynecologic malignancies are cervical cancer, ovarian cancer, and endometrial cancer, which pose a health threat to women around the world. The primary treatment for gynecological cancers is surgery, often accompanied by radiotherapy, chemotherapy, and immunotherapy. Some of these targeted medicines, like poly adenosine diphosphate ribose polymerase (PARP) inhibitors, have achieved considerable clinical effectiveness in ovarian cancer. Despite this, the generally disappointing prognosis for patients with gynecological tumors and there are still enormous obstacles that require more advancements in therapeutic approaches.^185^ Therefore, pyroptosis is an excellent starting point.

#### 4.7.1 Endometrial cancer

Endometrial cancer is one of the most prevalent gynecological cancers in women. Its incidence progressively climbed over 30 years, increasing by 132% overall.^186^ The three primary risk factors for endometrial cancer are hypertension, obesity, and diabetes. In this way, endometrial cancer may be classified as a metabolic disorder.^187^ Inflammation may be activated and promote tumor growth as well as invasion in endometrial cancer patients due to oxidative stress and elevated systemic inflammation produced by the metabolic syndrome.^188^ Endometrial cancer and inflammation are strongly associated, and pyroptosis and inflammation are inextricably linked. This makes it very interesting to explore the connection between pyroptosis and endometrial cancer.

In diabetic endometrial cancer patients, hexokinase domain‐containing 1 (HKDC1) is upregulated. HKDC1 causes pyroptosis and metabolic advantage in lactate‐rich environments by boosting ROS and glycolysis activation, resulting in the creation of acidic TME and favorable inflammatory that leads to endometrial cancer cell proliferation and migration. In terms of mechanisms, pyroptosis is controlled by the HOXC‐AS2/miR‐876‐5p/HKDC1 signaling axis.^189^ The study discovered that estrogen activates the NLPR3 inflammasome, triggering increased expression of NLPR3, ERβ, pro‐IL‐1β, IL‐1β, and endometrial cancer cell proliferation. Endometrial cancer progression and poor survival were associated with the upregulation of NLRP3, ASC, caspase 1, and IL‐1β.^190^ Another study reveals the opposite that hydrogen pretreatment suppresses endometrial cancer growth by raising ROS and pyroptosis‐related protein expression.^191^ Liang et al.^192^ evaluated nine pyroptosis‐related lncRNAs in the TCGA database and established a risk model that could be applied to forecast prognosis and assess response to immunotherapy. Zhang et al.^193^ identified four prognosis‐associated PRG (ELANE, GPX4, GSDMD, and TIRAP). This panel is also capable of forecasting the immune microenvironment and prognosis of endometrial cancer.

#### 4.7.2 Cervical cancer

There is no doubt that cervical cancer threatens women's health, as it is the fourth most common cause of death and incidence among women.^194^ Human papillomavirus (HPV) infection, particularly the type 16 and 18, exacerbates cervical cancer risk. Despite increasing HPV vaccination coverage, the treatment of cervical cancer resulting from HPV infection remains a clinical challenge.^195^ In HPV‐infected cervical cancer cells, sirtuin 1 (SIRT1) is overexpressed and represses NF‐κB‐driven transcription of the AIM2 gene along with pyroptotic death signaling.^196^ According to studies, HPV E7 reduces the self‐evasion from immune surveillance, induced cell pyroptosis, inflammasome activation, and generation of IL‐18 and IL‐1β by interacting with the E3 ligase TRIM21 to ubiquitinate and degrade the IFI16 inflammasome.^196^ According to the study, cells expressing the E6 oncogene could avoid immune surveillance by downregulating the expression of IL‐18 and preventing downstream cell damage because the HPV16 E6 lowered IL‐18 expression via an independent pathway of p53 degradation.^197^ Cell viability was substantially decreased and caspase 3/GSDME‐dependent pyroptosis was elevated in cervical cancer cells after lobaplatin treatment.^198^ The metal complex chemotherapeutic agent ruthenium (II) polypyridyl complex, Δ‐[Ru(bpy)2(HPIP)] (ClO4)2(Δ‐Ru1), is effective and safe enough to replace platinum drugs in the future. Taxol and Δ‐Ru1 inhibit tumor development and adverse effects via caspase 1/GSDMD‐dependent pyroptosis.^199^ Tanshinone II A, which may target the protein kinase domains of VEGF/VEGFR2,^200^ was reported to limit cell proliferation and increased pyroptosis in HeLa cells by upregulating the expression of miR‐145 and GSDMD.^201^ Meanwhile, overexpression of miR‐214 might facilitate pyroptosis and inhibit proliferation by targeting NLRP3 in cervical cancer.^202^ CHMP4C, GZMB, and TNF were identified in cervical cancer as differentially expressed pyroptosis‐related genes by Hu et al.,^203^ which can be a predictor for the outcome of cervical cancer.

#### 4.7.3 Ovarian cancer

Ovarian cancer, one of the most common gynecological malignancies, has a high mortality rate and unfavorable prognoses.^204^ In their TMEs, ovarian cancer patients have greater GSDMD and lower GSDME protein levels.^205^ Serous ovarian cancer has higher expression of GSDMD and GSDMC, compared with lower levels of GSDME and PJVK. In the genes that code for GSDMC, GSDMD, GSDME, and PJVK, ovarian cancer has the highest rate of copy number variation events of any cancer type.^206^ A disequilibrium of phosphatidylinositol and cholesterol in the cell membrane hinders GSDM integration and pores formation.^207^ Given the prevalence of this imbalance in malignancies, the hypothesis is that higher levels of phosphatidylinositide and cholesterol in the cell membrane keep cancer cells alive.^208^ This imbalance is usual in cancers, particularly ovarian cancer. Pyroptosis‐restricting metabolic changes are necessary for cancer cell survival.^209^ Li et al.^210^ found that IL‐1α and IL‐1β were overexpressed in a 1992 investigation on epithelial tumor cell lines and tumor cells derived from the ascitic fluid of ovarian cancer patients. In ovarian cancer cells with the 185delAG founder mutation in the breast cancer susceptibility gene 1, there was an increase in active IL‐1 and elevation in caspase 1 cleavage mediated by inflammasome.^211^ Olaparib, a PARP inhibitor, exhibits considerable cytotoxic effects and safety when combined with an IL‐1β inhibitor.^212^

NK cell‐mediated immunity removes ovarian cancer via cytokine‐induced memory‐like NK cells, which generate IFN‐γ and TNF‐α after seven days of IL‐18 exposure.^213^ When coupled with pegylated liposomal DOX, the immunostimulatory cytokine recombinant IL‐18 (SB‐485232) displays anticancer activity in preclinical models. Moreover, it has been proven to be safe and therapeutically effective in Phase I clinical trials in recurrent ovarian cancer.^214^ NLRP3 is involved in ovarian cancer chemoresistance with downregulation increasing drug sensitivity in gemcitabine‐resistant cell lines and overexpression inducing IL‐1β, EMT, and Wnt/β‐catenin signaling.^215^ Carboplatin therapy increases the activation of the NLRP3 inflammasome in macrophages, indicating NLRP3 implications for ovarian cancer and potential chemoresistance.^216^

NLRP1 inflammasome‐induced pyroptosis was exacerbated by the inhibition of lncRNA HOTTIP, which suppressed ovarian cancer.^217^ lncRNA GAS5 prevents the progression of ovarian cancer by activating ASC, caspase 1, and IL‐1 in a time‐dependent manner to create inflammasomes and induce pyroptosis.^218^ Additionally, one of the eight pyroptosis‐related lncRNAs, lncRNA (DICER1‐AS1), drastically suppressed the proliferation of ovarian cancer cells.^219^ Inhibition of fos‐like antigen 2 (FOSL2) limits ovarian cancer development through upregulation of proteins associated with inflammasome formation, including ASC, pro‐caspase 1, caspase 1, pro‐IL‐1, pro‐IL‐18, and IL‐18.^220^ The latest small molecule inhibitor, BI 2536, inhibits the proliferation of ovarian cancer cells, triggers cell cycle halt at the G2/M phase, and accumulates CD8+ T lymphocyte cells at tumor sites via initiating pyroptosis in ovarian cancer through the caspase 3/GSDME pathway.^43^ Bexarotene, a retinoid X receptor‐selective agonist,^221^ provokes pyroptotic cell death in an ovarian cancer cell line by a caspase 4‐GSDME‐dependent signaling cascade.^222^ NOB, a polymethoxyflavonoid, stimulates traditional autophagy, ROS production, and a decline in MMP, all of which contribute to initiating GSDMD/GSDME‐mediated pyroptosis and upregulating the expression of IL‐1β and ASC in ovarian cancer.^223^ Osthole, a potential inhibitor of histamine H1 receptor activity,^224^ also accelerates autophagy, apoptosis, and GSDME‐dependent pyroptosis while raising ROS generation and diminishing MMP.^225^ A new drug known as 2‐(anaphthoyl)ethyltrimethylammonium iodide (α‐NETA), a choline acetyltransferase inhibitor,^226^ hinders the development of epithelial ovarian cancer by activating pyroptosis via the GSDMD/caspase 4 pathway.^227^ The caspase 4/TXNIP–NLRP3–GSDMD pathway increases pyroptosis in response to treatment with citric acid for ovarian cancer.^228^

In addition to therapeutic applications, pyroptosis in ovarian cancer has prognostic applications. Antiangiogenic therapy effectiveness in ovarian cancer is predicted by AIM2. Higher levels of AIM2 in bevacizumab‐resistant patients are related to poorer progression‐free survival.^229^ Greater NLRP3 expression was related to worse overall survival in ovarian cancer.^230^ Cao et al.^219^ investigated the mutation frequency of NLRP3 was explored to have the maximum mutation frequency among the 33 PRGs. In ovarian cancer and healthy tissues, Qi and coworkers^231^ identified 31 distinct PRGs, with 13 downregulated and 18 upregulated. They established prognostic models and risk patterns. Another research employed 51 PRGs to construct the Pyrsig score, which clarified the immunological and prognostic relevance of ovarian cancer pyroptosis.^232^ The mechanisms and effects of pyroptosis‐related targets and agents in gynecological oncology are summarized in Table 2.

**FIGURE 4:**
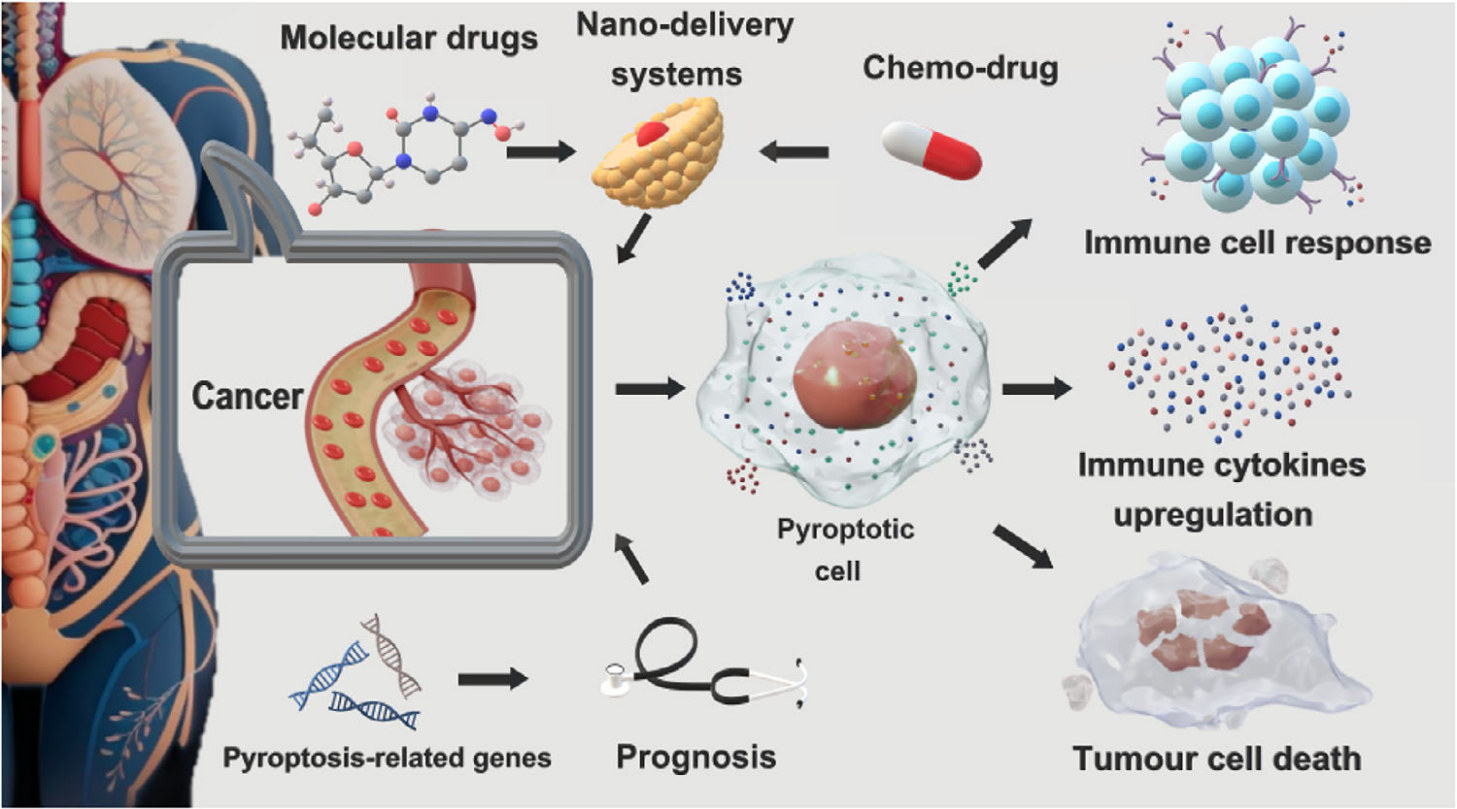
Prospects for pyroptosis‐related targeted therapy in cancer. Molecular drugs and chemo‐drugs loaded into the nanodelivery system to target cancer cells and activate pyroptosis, resulting in immune cell response, elevated immune cytokines and tumor cell death.

We summarized the relevant applications of pyroptosis in cancer in Figure 4. Personalized treatment approaches will be achieved by exploring the possibility of targeting specific pyroptosis pathways in different types of cancer. In addition, understanding the interactions between pyroptosis and other immune cell death pathways may provide valuable insights into novel therapeutic strategies for cancer patients.

## 5 PYROPTOSIS AND INFLAMMASOMES IN INFLAMMATION

Pyroptosis, mediated by inflammasomes, is a highly inflammatory form of cell death that plays a crucial role in the body's immune response.  Pyroptosis and inflammasomes are responsible for processing inflammatory cytokines and initiating an inflammatory cascade. Understanding the association between pyroptosis, inflammasomes, and inflammatory diseases is essential for curing inflammatory diseases.

### 5.1 Involvement of pyroptosis and inflammasomes in inflammatory diseases

Pyroptosis and inflammasomes have been implicated in various inflammatory diseases. For example, in rheumatoid arthritis, the assembly of NLRP3 plays a crucial role in activating immune cells and releasing IL‐1β and IL‐18, leading to joint inflammation and damage.^233^ Targeting these inflammasomes could potentially alleviate symptoms and slow down disease progression. Similarly, in Crohn's disease, GSDME‐mediated pyroptosis to release proinflammatory cytokines in epithelial cells can contribute to tissue damage and chronic inflammation.^234^ It has been discovered that IL‐18 levels are higher in Crohn's disease patients and that it contributes to the promotion of TH1 cell responses.^235^ Furthermore, recent studies have shown that dysregulation of pyroptosis and inflammasome activation can also play a role in the pathogenesis of cardiovascular diseases, such as atherosclerosis and myocardial infarction.^236^, ^237^ There is evidence that plaques and ruptured lesions linked to acute coronary events in atherosclerosis exhibit large elevations in caspase 1 expression.^238^ Additionally, the activation of inflammasomes has been implicated in the development and progression of neurodegenerative disorders, such as Alzheimer's disease and Parkinson's disease.^239^ Therefore, targeting pyroptosis and inflammasomes may offer promising therapeutic opportunities for a wide range of inflammatory diseases beyond the traditionally studied conditions.

### 5.2 Molecular mechanisms linking pyroptosis and inflammation

IL‐1β and IL‐18 are two proinflammatory cytokines that are directly produced during pyroptosis. Both cytokines have key roles in the pathophysiology of a spectrum of inflammatory disorders. Vasodilation, fever, hematopoiesis, leukocyte infiltration, antibody generation, and the expression of cytokines and chemokines are all events triggered by IL‐1β binding to IL‐1 receptors on the surface of immune cells.^9^ A key factor in immunological responses, IL‐18 is involved in angiogenesis and the induction of IFNγ, the activation of T cells, macrophages, and NK cells.^240^, ^241^ In general, pyroptosis is beneficial to the host because it fights infections and cellular stress. Despite these two proinflammatory cytokines are known to be produced directly by inflammasome activation, other cytokines, such as TNF‐ β, IL‐6, and IL‐16,^242^, ^243^ are indirectly produced, which can cause an inflammatory storm and tissue damage. Additionally, it was shown that the IFNγ and TNFα boosted the proinflammatory response and triggered pyroptosis.^244^ By activating the inflammasome, proinflammatory cytokines can be made, which can cause an inflammatory storm and damage to tissues. This can make pyroptosis less effective at fighting infections and cellular stress; it could even cause damage. More research is needed to understand the role of the inflammasome and pyroptosis in inflammatory diseases. By developing inhibitors or agonists that target the pyroptosis pathway and lead to a therapeutic effect, researchers hope to find new treatments for inflammatory diseases.

### 5.3 Therapeutic implications of targeting pyroptosis and inflammasomes in inflammation

Targeting pyroptosis and inflammasomes in inflammation diseases could be used as a way to treat inflammation by going after the NLRP3 and GSDMD proteins. Some of these inhibitors are already adopted in clinical trials. MCC950, a nanomolar potent and specific inhibitor of NLRP3 signaling, was active in several models of inflammatory disease.^245^ MCC950 inhibits the ATPase activity of NLRP3 and also attaches to the NACTH domain, trapping NLRP3 in an inactive state.^68^, ^246^ Punicalagin, which affects plasma membrane fluidity, may have implications for its potential role in modulating N‐GSDMD insertion or oligomerization.^247^

## 6 CONCLUSION AND PERSPECTIVES

Pyroptosis is a specific form of PCD. Activation of inflammasomes is a crucial factor in inducing pyroptosis. In this review, we describe the mechanisms of pyroptosis and activation of inflammasomes and their applications in cancer and inflammatory diseases. Pyroptosis leads to the secretion of proinflammatory cytokines, which not only contribute to inflammation but also play a crucial role in the immune response against cancer cells. Therefore, targeting the pyroptosis pathway using nanomedicines and inhibitors holds great potential for therapeutic interventions in both cancer and inflammatory diseases, and we therefore summarize this review. Pyroptosis‐related genes may aid in predicting the prognosis and treatment efficacy of tumors. These genes have been linked to tumor progression and therapeutic response. Besides, their levels of expression can serve as potential biomarkers in personalized cancer treatment strategies. However, additional in‐depth research and clinical applications are required since the understanding of pyroptosis‐related genes in tumor biology is continually evolving. Further studies are needed to determine the exact mechanisms through which pyroptosis‐related genes impact tumor growth and treatment response. Beyond that, clinical trials are needed to validate the efficacy of these genes as predictive biomarkers in cancer patients, which would facilitate the development of personalized therapeutics. The use of PRG as a new and accurate diagnostic and prognostic for tumors is anticipated in the future.

Pyroptosis has an essential effect on the growth and chemotherapeutic treatment resistance of tumors because GSDME has a great impact on the response to chemotherapy or immunotherapy. Low levels of GSDME, generated by hypermethylation of the GSDME mRNA in tumor cells, impair the ability of most tumor cells to undergo pyroptosis and stimulate tumor growth. This interaction may enhance the resistance of tumor cells to chemotherapeutic drugs and hinder the effectiveness of immunotherapy. Additionally, the response to these treatments may be improved by targeting GSDME methylation and restoring the ability of tumor cells to undergo pyroptosis and restrain tumor development. For instance, DAC can demethylate GSDME, restoring normal expression of the GSDME protein and boosting the effectiveness of antitumor treatment. In addition, certain inhibitors and drugs, like those that activate the caspase 1/GSDMD or caspase 3/GSDME pathway, can cause the onset of pyroptosis. These agents can effectively eliminate cancer cells and prevent tumor growth by provoking pyroptosis in tumor cells. As a result, it has great potential for improving the outcomes of antitumor therapy by utilizing pyroptosis‐inducing agents, especially those that target GSDME methylation.

Pyroptosis can be anticancer or procancer, but in most cases, it is a cancer suppressor in most types of cancer, although its function in cancer is not fully understood. Therefore, the following therapeutic recommendations will promote the process of pyroptosis in cancer. There is also an unavoidable problem. The fatal CRS might occur after pyroptosis therapy as an inflammatory way for cells to die, injuring surrounding or other normal tissues and creating a range of additional problems. Researchers are experimenting with several strategies to mitigate the risks associated with CRS during pyroptosis therapy. One approach is to create targeted delivery systems that can specifically induce pyroptosis in cancer cells while sparing healthy tissues. Moreover, efforts are conducted to identify biomarkers or molecular signatures that may forecast the probability of CRS development, enabling the plan of customized treatment programs and more thorough monitoring of patients.

Although inducing substances may evoke pyroptosis, they have considerable systemic adverse effects because they are insoluble, nonspecific, and disintegrate fast in vivo. Nanodelivery systems can boost the efficacy of pyroptosis‐inducing agents by improving their stability and specificity. These systems can also distribute the agents in a regulated manner, allowing for continuous and targeted delivery to cancer cells. Meanwhile, nanodelivery systems can overcome the limitations of insolubility and fast degradation, making them a valuable tool in cancer treatment. Many investigations on nanomedicine for tumors in the field of pyroptosis have been conducted. These studies have yielded encouraging findings in terms of increased cancer cell death and decreased toxicity to healthy cells. Nevertheless, most of them have never been used in clinical practice. The challenges of scaling up production and ensuring consistent quality control are linked to the limited usage of nanomedicines in clinical practice. On the other hand, the expensive cost of developing and manufacturing nanomedicine products may impede their broad use in healthcare settings. Therefore, further research and clinical trials are required to demonstrate the efficacy and safety of nanomedicines, paving the way for their widespread adoption and incorporation into healthcare systems.

Certain pyroptosis inflammatory factors can lead to immune cell responses that modify the TME and have a procancer or anticancer effect. There are few investigations on this subject because the use of pyroptosis‐associated inflammatory factors for cancer therapy is still in its early phases. The inflammasome appears to be a promising treatment target as well as a valuable biomarker for cancer. It could potentially lead to the development of targeted therapies that exploit its procancer or anticancer effects if the function of the inflammasome in cancer is understood. Further research into the use of pyroptosis‐associated inflammatory factors as prospective therapeutic alternatives may provide insights into improving the effectiveness of cancer treatment. These studies will concentrate on the involvement of the inflammasome in cancer as well as the application of particular inflammasome‐related treatments in clinical and preclinical trials.

Understanding the mechanisms of pyroptosis and inflammasomes in disease contexts is crucial for developing targeted therapeutic strategies. By targeting these pathways, we may be able to modulate immune responses and inflammation, leading to improved outcomes in cancer or inflammatory diseases. Further research in this area is required to fully comprehend the complex interplay between pyroptosis, inflammasomes, and disease progression, which could pave the way for innovative treatments and personalized medicine approaches.

## AUTHOR CONTRIBUTIONS

J. W. and S. H. are responsible for the collection of data, collation, and writing of the original manuscript. H. B. and Y. J. are responsible for reviewing the literature. S. C. and Y. Z. are responsible for the concept development, revision, and review of the manuscript. All authors contributed to the article and approved the submitted version.

## CONFLICT OF INTEREST STATEMENT

The authors declare that the research was conducted in the absence of any commercial or financial relationships that could be construed as a potential conflict of interest. The authors declare that they have no conflicts of interest pertaining to this study.

## ETHICS STATEMENT

Not applicable.

## Data Availability

Not applicable.

## References

[1] Su Z , Yang Z , Xie L , DeWitt JP , Chen Y . Cancer therapy in the necroptosis era. Cell Death Differ. 2016;23(5):748‐756. 26915291 10.1038/cdd.2016.8 PMC4832112

[2] Zychlinsky A , Prevost MC , Sansonetti PJ . Shigella flexneri induces apoptosis in infected macrophages. Nature. 1992;358(6382):167‐169. 1614548 10.1038/358167a0

[3] Chen GY , Nuñez G . Sterile inflammation: sensing and reacting to damage. Nat Rev Immunol. 2010;10(12):826‐837. 21088683 10.1038/nri2873 PMC3114424

[4] Guo H , Callaway JB , Ting JP . Inflammasomes: mechanism of action, role in disease, and therapeutics. Nat Med. 2015;21(7):677‐687. 26121197 10.1038/nm.3893 PMC4519035

[5] Hou L , Yang Z , Wang Z , et al. NLRP3/ASC‐mediated alveolar macrophage pyroptosis enhances HMGB1 secretion in acute lung injury induced by cardiopulmonary bypass. Lab Invest. 2018;98(8):1052‐1064. 29884910 10.1038/s41374-018-0073-0

[6] Shi J , Zhao Y , Wang K , et al. Cleavage of GSDMD by inflammatory caspases determines pyroptotic cell death. Nature. 2015;526(7575):660‐665. 26375003 10.1038/nature15514

[7] Guo B , Fu S , Zhang J , Liu B , Li Z . Targeting inflammasome/IL‐1 pathways for cancer immunotherapy. Sci Rep. 2016;6:36107. 27786298 10.1038/srep36107 PMC5082376

[8] Siegel RL , Miller KD , Fuchs HE , Jemal A . Cancer statistics, 2022. CA Cancer J Clin. 2022;72(1):7‐33. 35020204 10.3322/caac.21708

[9] Delaleu N , Bickel M . Interleukin‐1 beta and interleukin‐18: regulation and activity in local inflammation. Periodontol. 2004;35:42‐52. 2000. 10.1111/j.0906-6713.2004.003569.x 15107057

[10] Evavold CL , Ruan J , Tan Y , Xia S , Wu H , Kagan JC . The pore‐forming protein gasdermin D regulates interleukin‐1 secretion from living macrophages. Immunity. 2018;48(1):35‐44.e6. 29195811 10.1016/j.immuni.2017.11.013 PMC5773350

[11] Cookson BT , Brennan MA . Pro‐inflammatory programmed cell death. Trends Microbiol. 2001;9(3):113‐114. 11303500 10.1016/s0966-842x(00)01936-3

[12] Hilbi H , Chen Y , Thirumalai K , Zychlinsky A . The interleukin 1beta‐converting enzyme, caspase 1, is activated during Shigella flexneri‐induced apoptosis in human monocyte‐derived macrophages. Infect Immun. 1997;65(12):5165‐5170. 9393811 10.1128/iai.65.12.5165-5170.1997 PMC175744

[13] Hersh D , Monack DM , Smith MR , Ghori N , Falkow S , Zychlinsky A . The Salmonella invasin SipB induces macrophage apoptosis by binding to caspase‐1. Proc Natl Acad Sci USA. 1999;96(5):2396‐2401. 10051653 10.1073/pnas.96.5.2396 PMC26795

[14] Martinon F , Burns K , Tschopp J . The inflammasome: a molecular platform triggering activation of inflammatory caspases and processing of proIL‐beta. Mol Cell. 2002;10(2):417‐426. 12191486 10.1016/s1097-2765(02)00599-3

[15] Ding J , Wang K , Liu W , et al. Pore‐forming activity and structural autoinhibition of the gasdermin family. Nature. 2016;535(7610):111‐116. 27281216 10.1038/nature18590

[16] Wang Y , Gao W , Shi X , et al. Chemotherapy drugs induce pyroptosis through caspase‐3 cleavage of a gasdermin. Nature. 2017;547(7661):99‐103. 28459430 10.1038/nature22393

[17] Galluzzi L , Vitale I , Aaronson SA , et al. Molecular mechanisms of cell death: recommendations of the Nomenclature Committee on Cell Death 2018. Cell Death Differ. 2018;25(3):486‐541. 29362479 10.1038/s41418-017-0012-4 PMC5864239

[18] Rathinam VA , Vanaja SK , Waggoner L , et al. TRIF licenses caspase‐11‐dependent NLRP3 inflammasome activation by gram‐negative bacteria. Cell. 2012;150(3):606‐619. 22819539 10.1016/j.cell.2012.07.007 PMC3660860

[19] Kayagaki N , Warming S , Lamkanfi M , et al. Non‐canonical inflammasome activation targets caspase‐11. Nature. 2011;479(7371):117‐121. 22002608 10.1038/nature10558

[20] Zhang JY , Zhou B , Sun RY , et al. The metabolite α‐KG induces GSDMC‐dependent pyroptosis through death receptor 6‐activated caspase‐8. Cell Res. 2021;31(9):980‐997. 34012073 10.1038/s41422-021-00506-9 PMC8410789

[21] Voskoboinik I , Whisstock JC , Trapani JA . Perforin and granzymes: function, dysfunction and human pathology. Nat Rev Immunol. 2015;15(6):388‐400. 25998963 10.1038/nri3839

[22] Zheng D , Liwinski T , Elinav E . Inflammasome activation and regulation: toward a better understanding of complex mechanisms. Cell Discov. 2020;6:36. 32550001 10.1038/s41421-020-0167-x PMC7280307

[23] Wu J , Fernandes‐Alnemri T , Alnemri ES . Involvement of the AIM2, NLRC4, and NLRP3 inflammasomes in caspase‐1 activation by Listeria monocytogenes. J Clin Immunol. 2010;30(5):693‐702. 20490635 10.1007/s10875-010-9425-2 PMC3321545

[24] Schnappauf O , Chae JJ , Kastner DL , Aksentijevich I . The pyrin inflammasome in health and disease. Front Immunol. 2019;10:1745. 31456795 10.3389/fimmu.2019.01745 PMC6698799

[25] Lu A , Magupalli VG , Ruan J , et al. Unified polymerization mechanism for the assembly of ASC‐dependent inflammasomes. Cell. 2014;156(6):1193‐1206. 24630722 10.1016/j.cell.2014.02.008 PMC4000066

[26] Zhao Y , Yang J , Shi J , et al. The NLRC4 inflammasome receptors for bacterial flagellin and type III secretion apparatus. Nature. 2011;477(7366):596‐600. 21918512 10.1038/nature10510

[27] Sandstrom A , Mitchell PS , Goers L , Mu EW , Lesser CF , Vance RE . Functional degradation: a mechanism of NLRP1 inflammasome activation by diverse pathogen enzymes. Science. 2019;364(6435). 10.1126/science.aau1330 PMC6532986 30872533

[28] de Alba E . Structure and interdomain dynamics of apoptosis‐associated speck‐like protein containing a CARD (ASC). J Biol Chem. 2009;284(47):32932‐32941. 19759015 10.1074/jbc.M109.024273 PMC2781708

[29] Broz P , Dixit VM . Inflammasomes: mechanism of assembly, regulation and signalling. Nat Rev Immunol. 2016;16(7):407‐420. 27291964 10.1038/nri.2016.58

[30] Boucher D , Monteleone M , Coll RC , et al. Caspase‐1 self‐cleavage is an intrinsic mechanism to terminate inflammasome activity. J Exp Med. 2018;215(3):827‐840. 29432122 10.1084/jem.20172222 PMC5839769

[31] Xia S , Zhang Z , Magupalli VG , et al. Gasdermin D pore structure reveals preferential release of mature interleukin‐1. Nature. 2021;593(7860):607‐611. 33883744 10.1038/s41586-021-03478-3 PMC8588876

[32] Panganiban RA , Sun M , Dahlin A , et al. A functional splice variant associated with decreased asthma risk abolishes the ability of gasdermin B to induce epithelial cell pyroptosis. J Allergy Clin Immunol. 2018;142(5):1469‐1478. e2 29330013 10.1016/j.jaci.2017.11.040 PMC6037620

[33] Matikainen S , Nyman TA , Cypryk W . Function and regulation of noncanonical caspase‐4/5/11 inflammasome. J Immunol. 2020;204(12):3063‐3069. 32513874 10.4049/jimmunol.2000373

[34] Man SM , Place DE , Kuriakose T , Kanneganti TD . Interferon‐inducible guanylate‐binding proteins at the interface of cell‐autonomous immunity and inflammasome activation. J Leukoc Biol. 2017;101(1):143‐150. 27418355 10.1189/jlb.4MR0516-223R PMC6608036

[35] Shi J , Zhao Y , Wang Y , et al. Inflammatory caspases are innate immune receptors for intracellular LPS. Nature. 2014;514(7521):187‐192. 25119034 10.1038/nature13683

[36] Kayagaki N , Stowe IB , Lee BL , et al. Caspase‐11 cleaves gasdermin D for non‐canonical inflammasome signalling. Nature. 2015;526(7575):666‐671. 26375259 10.1038/nature15541

[37] Yang D , He Y , Munoz‐Planillo R , Liu Q , Nunez G . Caspase‐11 requires the pannexin‐1 channel and the purinergic p2×7 pore to mediate pyroptosis and endotoxic shock. Immunity. 2015;43(5):923‐932. 26572062 10.1016/j.immuni.2015.10.009 PMC4795157

[38] Rühl S , Broz P . Caspase‐11 activates a canonical NLRP3 inflammasome by promoting K(+) efflux. Eur J Immunol. 2015;45(10):2927‐2936. 26173909 10.1002/eji.201545772

[39] Schmid‐Burgk JL , Gaidt MM , Schmidt T , Ebert TS , Bartok E , Hornung V . Caspase‐4 mediates non‐canonical activation of the NLRP3 inflammasome in human myeloid cells. Eur J Immunol. 2015;45(10):2911‐2917. 26174085 10.1002/eji.201545523

[40] Chen Q , Shi P , Wang Y , et al. GSDMB promotes non‐canonical pyroptosis by enhancing caspase‐4 activity. J Mol Cell Biol. 2019;11(6):496‐508. 30321352 10.1093/jmcb/mjy056 PMC6734491

[41] Rogers C , Fernandes‐Alnemri T , Mayes L , Alnemri D , Cingolani G , Alnemri ES . Cleavage of DFNA5 by caspase‐3 during apoptosis mediates progression to secondary necrotic/pyroptotic cell death. Nat Commun. 2017;8:14128. 28045099 10.1038/ncomms14128 PMC5216131

[42] Tan Y , Chen Q , Li X , et al. Pyroptosis: a new paradigm of cell death for fighting against cancer. J Exp Clin Cancer Res. 2021;40(1):153. 33941231 10.1186/s13046-021-01959-x PMC8091792

[43] Huo J , Shen Y , Zhang Y , Shen L . BI 2536 induces gasdermin E‐dependent pyroptosis in ovarian cancer. Front Oncol. 2022;12:963928. 36016611 10.3389/fonc.2022.963928 PMC9396031

[44] Tan G , Huang C , Chen J , Zhi F . HMGB1 released from GSDME‐mediated pyroptotic epithelial cells participates in the tumorigenesis of colitis‐associated colorectal cancer through the ERK1/2 pathway. J Hematol Oncol. 2020;13(1):149. 33160389 10.1186/s13045-020-00985-0 PMC7648939

[45] Chao KL , Kulakova L , Herzberg O . Gene polymorphism linked to increased asthma and IBD risk alters gasdermin‐B structure, a sulfatide and phosphoinositide binding protein. Proc Natl Acad Sci USA. 2017;114(7):E1128‐E1137. 28154144 10.1073/pnas.1616783114 PMC5321033

[46] Kalkavan H , Green DR . MOMP, cell suicide as a BCL‐2 family business. Cell Death Differ. 2018;25(1):46‐55. 29053143 10.1038/cdd.2017.179 PMC5729535

[47] Wang YY , Liu XL , Zhao R . Induction of pyroptosis and its implications in cancer management. Front Oncol. 2019;9:971. 31616642 10.3389/fonc.2019.00971 PMC6775187

[48] Yu P , Wang HY , Tian M , et al. Eukaryotic elongation factor‐2 kinase regulates the cross‐talk between autophagy and pyroptosis in doxorubicin‐treated human melanoma cells in vitro. Acta Pharmacol Sin. 2019;40(9):1237‐1244. 30914761 10.1038/s41401-019-0222-z PMC6786479

[49] Zhang CC , Li CG , Wang YF , et al. Chemotherapeutic paclitaxel and cisplatin differentially induce pyroptosis in A549 lung cancer cells via caspase‐3/GSDME activation. Apoptosis. 2019;24(3‐4):312‐325. 30710195 10.1007/s10495-019-01515-1

[50] Zeng CY , Li CG , Shu JX , et al. ATP induces caspase‐3/gasdermin E‐mediated pyroptosis in NLRP3 pathway‐blocked murine macrophages. Apoptosis. 2019;24(9‐10):703‐717. 31175486 10.1007/s10495-019-01551-x

[51] Sarhan J , Liu BC , Muendlein HI , et al. Caspase‐8 induces cleavage of gasdermin D to elicit pyroptosis during Yersinia infection. Proc Natl Acad Sci USA. 2018;115(46):E10888‐E10897. 30381458 10.1073/pnas.1809548115 PMC6243247

[52] Hou J , Zhao R , Xia W , et al. PD‐L1‐mediated gasdermin C expression switches apoptosis to pyroptosis in cancer cells and facilitates tumour necrosis. Nat Cell Biol. 2020;22(10):1264‐1275. 32929201 10.1038/s41556-020-0575-z PMC7653546

[53] Ewen CL , Kane KP , Bleackley RC . A quarter century of granzymes. Cell Death Differ. 2012;19(1):28‐35. 22052191 10.1038/cdd.2011.153 PMC3252830

[54] Heusel JW , Wesselschmidt RL , Shresta S , Russell JH , Ley TJ . Cytotoxic lymphocytes require granzyme B for the rapid induction of DNA fragmentation and apoptosis in allogeneic target cells. Cell. 1994;76(6):977‐987. 8137431 10.1016/0092-8674(94)90376-x

[55] Zhou Z , He H , Wang K , et al. Granzyme A from cytotoxic lymphocytes cleaves GSDMB to trigger pyroptosis in target cells. Science. 2020;368(6494). 10.1126/science.aaz7548 32299851

[56] Feng S , Fox D , Man SM . Mechanisms of gasdermin family members in inflammasome signaling and cell death. J Mol Biol. 2018;430(18):3068‐3080. 29990470 10.1016/j.jmb.2018.07.002

[57] Zhang Z , Zhang Y , Xia S , et al. Gasdermin E suppresses tumour growth by activating anti‐tumour immunity. Nature. 2020;579(7799):415‐420. 32188940 10.1038/s41586-020-2071-9 PMC7123794

[58] Liu X , Zhang Z , Ruan J , et al. Inflammasome‐activated gasdermin D causes pyroptosis by forming membrane pores. Nature. 2016;535(7610):153‐158. 27383986 10.1038/nature18629 PMC5539988

[59] Sborgi L , Rühl S , Mulvihill E , et al. GSDMD membrane pore formation constitutes the mechanism of pyroptotic cell death. EMBO J. 2016;35(16):1766‐1778. 27418190 10.15252/embj.201694696 PMC5010048

[60] de Vasconcelos NM , Van Opdenbosch N , Van Gorp H , Parthoens E , Lamkanfi M . Single‐cell analysis of pyroptosis dynamics reveals conserved GSDMD‐mediated subcellular events that precede plasma membrane rupture. Cell Death Differ. 2019;26(1):146‐161. 29666477 10.1038/s41418-018-0106-7 PMC6294780

[61] Franchi L , Warner N , Viani K , Nuñez G . Function of Nod‐like receptors in microbial recognition and host defense. Immunol Rev. 2009;227(1):106‐128. 19120480 10.1111/j.1600-065X.2008.00734.x PMC2679989

[62] Finger JN , Lich JD , Dare LC , et al. Autolytic proteolysis within the function to find domain (FIIND) is required for NLRP1 inflammasome activity. J Biol Chem. 2012;287(30):25030‐25037. 22665479 10.1074/jbc.M112.378323 PMC3408201

[63] Hornung V , Ablasser A , Charrel‐Dennis M , et al. AIM2 recognizes cytosolic dsDNA and forms a caspase‐1‐activating inflammasome with ASC. Nature. 2009;458(7237):514‐518. 19158675 10.1038/nature07725 PMC2726264

[64] Yu JW , Fernandes‐Alnemri T , Datta P , et al. Pyrin activates the ASC pyroptosome in response to engagement by autoinflammatory PSTPIP1 mutants. Mol Cell. 2007;28(2):214‐227. 17964261 10.1016/j.molcel.2007.08.029 PMC2719761

[65] Elliott EI , Sutterwala FS . Initiation and perpetuation of NLRP3 inflammasome activation and assembly. Immunol Rev. 2015;265(1):35‐52. 25879282 10.1111/imr.12286 PMC4400874

[66] Muñoz‐Planillo R , Kuffa P , Martínez‐Colón G , Smith BL , Rajendiran TM , Núñez G . K⁺ efflux is the common trigger of NLRP3 inflammasome activation by bacterial toxins and particulate matter. Immunity. 2013;38(6):1142‐1153. 23809161 10.1016/j.immuni.2013.05.016 PMC3730833

[67] Vajjhala PR , Mirams RE , Hill JM . Multiple binding sites on the pyrin domain of ASC protein allow self‐association and interaction with NLRP3 protein. J Biol Chem. 2012;287(50):41732‐41743. 23066025 10.1074/jbc.M112.381228 PMC3516722

[68] Tapia‐Abellán A , Angosto‐Bazarra D , Martínez‐Banaclocha H , et al. MCC950 closes the active conformation of NLRP3 to an inactive state. Nat Chem Biol. 2019;15(6):560‐564. 31086329 10.1038/s41589-019-0278-6 PMC7116292

[69] Franchi L , Eigenbrod T , Núñez G . Cutting edge: tNF‐alpha mediates sensitization to ATP and silica via the NLRP3 inflammasome in the absence of microbial stimulation. J Immunol. 2009;183(2):792‐796. 19542372 10.4049/jimmunol.0900173 PMC2754237

[70] Mitchell PS , Sandstrom A , Vance RE . The NLRP1 inflammasome: new mechanistic insights and unresolved mysteries. Curr Opin Immunol. 2019;60:37‐45. 31121538 10.1016/j.coi.2019.04.015 PMC6800612

[71] Chui AJ , Okondo MC , Rao SD , et al. N‐terminal degradation activates the NLRP1B inflammasome. Science. 2019;364(6435):82‐85. 30872531 10.1126/science.aau1208 PMC6610862

[72] Bauernfried S , Scherr MJ , Pichlmair A , Duderstadt KE , Hornung V . Human NLRP1 is a sensor for double‐stranded RNA. Science. 2021;371(6528). 10.1126/science.abd0811 33243852

[73] Xu H , Yang J , Gao W , et al. Innate immune sensing of bacterial modifications of Rho GTPases by the Pyrin inflammasome. Nature. 2014;513(7517):237‐241. 24919149 10.1038/nature13449

[74] Gavrilin MA , Abdelaziz DH , Mostafa M , et al. Activation of the pyrin inflammasome by intracellular Burkholderia cenocepacia. J Immunol. 2012;188(7):3469‐3477. 22368275 10.4049/jimmunol.1102272 PMC3482472

[75] Du T , Gao J , Li P , et al. Pyroptosis, metabolism, and tumor immune microenvironment. Clin Transl Med. 2021;11(8):e492. 34459122 10.1002/ctm2.492 PMC8329701

[76] Bauernfeind FG , Horvath G , Stutz A , et al. Cutting edge: nF‐kappaB activating pattern recognition and cytokine receptors license NLRP3 inflammasome activation by regulating NLRP3 expression. J Immunol. 2009;183(2):787‐791. 19570822 10.4049/jimmunol.0901363 PMC2824855

[77] Galluzzi L , Buqué A , Kepp O , Zitvogel L , Kroemer G . Immunogenic cell death in cancer and infectious disease. Nat Rev Immunol. 2017;17(2):97‐111. 27748397 10.1038/nri.2016.107

[78] Liang Q , Wu J , Zhao X , et al. Establishment of tumor inflammasome clusters with distinct immunogenomic landscape aids immunotherapy. Theranostics. 2021;11(20):9884‐9903. 34815793 10.7150/thno.63202 PMC8581407

[79] Fitzgerald AA , Wang S , Agarwal V , et al. DPP inhibition alters the CXCR3 axis and enhances NK and CD8+ T cell infiltration to improve anti‐PD1 efficacy in murine models of pancreatic ductal adenocarcinoma. J Immunother Cancer. 2021;9(11). 10.1136/jitc-2021-002837 PMC8578994 34737215

[80] Janowski AM , Colegio OR , Hornick EE , et al. NLRC4 suppresses melanoma tumor progression independently of inflammasome activation. J Clin Invest. 2016;126(10):3917‐3928. 27617861 10.1172/JCI86953 PMC5096827

[81] Corrales L , Woo SR , Williams JB , McWhirter SM , Dubensky TW , Gajewski TF . Antagonism of the STING pathway via activation of the AIM2 inflammasome by intracellular DNA. J Immunol. 2016;196(7):3191‐3198. 26927800 10.4049/jimmunol.1502538 PMC4800192

[82] Fukuda K , Okamura K , Riding RL , et al. AIM2 regulates anti‐tumor immunity and is a viable therapeutic target for melanoma. J Exp Med. 2021;218(9). 10.1084/jem.20200962 PMC8329870 34325468

[83] Shoham NG , Centola M , Mansfield E , et al. Pyrin binds the PSTPIP1/CD2BP1 protein, defining familial Mediterranean fever and PAPA syndrome as disorders in the same pathway. Proc Natl Acad Sci USA. 2003;100(23):13501‐13506. 14595024 10.1073/pnas.2135380100 PMC263843

[84] Park S , Cheon S , Cho D . The dual effects of interleukin‐18 in tumor progression. Cell Mol Immunol. 2007;4(5):329‐335. 17976312

[85] Bent R , Moll L , Grabbe S , Bros M . Interleukin‐1 beta‐A friend or foe in malignancies. Int J Mol Sci. 2018;19(8). 10.3390/ijms19082155 PMC6121377 30042333

[86] Li W , Wu K , Zhao E , et al. HMGB1 recruits myeloid derived suppressor cells to promote peritoneal dissemination of colon cancer after resection. Biochem Biophys Res Commun. 2013;436(2):156‐161. 23707808 10.1016/j.bbrc.2013.04.109

[87] Chen J , Liu X , Zhang J , Zhao Y . Targeting HMGB1 inhibits ovarian cancer growth and metastasis by lentivirus‐mediated RNA interference. J Cell Physiol. 2012;227(11):3629‐3638. 22331597 10.1002/jcp.24069

[88] Kuppala MB , Syed SB , Bandaru S , Varre S , Akka J , Mundulru HP . Immunotherapeutic approach for better management of cancer–role of IL‐18. Asian Pac J Cancer Prev. 2012;13(11):5353‐5361. 23317183 10.7314/apjcp.2012.13.11.5353

[89] Hutton HL , Ooi JD , Holdsworth SR , Kitching AR . The NLRP3 inflammasome in kidney disease and autoimmunity. Nephrology (Carlton). 2016;21(9):736‐744. 27011059 10.1111/nep.12785

[90] Sutton C , Brereton C , Keogh B , Mills KH , Lavelle EC . A crucial role for interleukin (IL)‐1 in the induction of IL‐17‐producing T cells that mediate autoimmune encephalomyelitis. J Exp Med. 2006;203(7):1685‐1691. 16818675 10.1084/jem.20060285 PMC2118338

[91] Williams TM , Leeth RA , Rothschild DE , et al. The NLRP1 inflammasome attenuates colitis and colitis‐associated tumorigenesis. J Immunol. 2015;194(7):3369‐3380. 25725098 10.4049/jimmunol.1402098 PMC4369420

[92] Ben‐Sasson SZ , Hogg A , Hu‐Li J , et al. IL‐1 enhances expansion, effector function, tissue localization, and memory response of antigen‐specific CD8 T cells. J Exp Med. 2013;210(3):491‐502. 23460726 10.1084/jem.20122006 PMC3600912

[93] Zhivaki D , Kagan JC . NLRP3 inflammasomes that induce antitumor immunity. Trends Immunol. 2021;42(7):575‐589. 34034975 10.1016/j.it.2021.05.001

[94] Tominaga K , Yoshimoto T , Torigoe K , et al. IL‐12 synergizes with IL‐18 or IL‐1beta for IFN‐gamma production from human T cells. Int Immunol. 2000;12(2):151‐160. 10653850 10.1093/intimm/12.2.151

[95] Lim HX , Hong HJ , Cho D , Kim TS . IL‐18 enhances immunosuppressive responses by promoting differentiation into monocytic myeloid‐derived suppressor cells. J Immunol. 2014;193(11):5453‐5460. 25362180 10.4049/jimmunol.1401282

[96] Dupaul‐Chicoine J , Arabzadeh A , Dagenais M , et al. The Nlrp3 inflammasome suppresses colorectal cancer metastatic growth in the liver by promoting natural killer cell tumoricidal activity. Immunity. 2015;43(4):751‐763. 26384545 10.1016/j.immuni.2015.08.013

[97] Strichman‐Almashanu LZ , Bustin M , Landsman D . Retroposed copies of the HMG genes: a window to genome dynamics. Genome Res. 2003;13(5):800‐812. 12727900 10.1101/gr.893803 PMC430908

[98] Li G , Liang X , Lotze MT . HMGB1: the central cytokine for all lymphoid cells. Front Immunol. 2013;4:68. 23519706 10.3389/fimmu.2013.00068 PMC3602962

[99] Li Z , Fu WJ , Chen XQ , et al. Autophagy‐based unconventional secretion of HMGB1 in glioblastoma promotes chemosensitivity to temozolomide through macrophage M1‐like polarization. J Exp Clin Cancer Res. 2022;41(1):74. 35193644 10.1186/s13046-022-02291-8 PMC8862393

[100] Yu M , Wang H , Ding A , et al. HMGB1 signals through toll‐like receptor (TLR) 4 and TLR2. Shock. 2006;26(2):174‐179. 16878026 10.1097/01.shk.0000225404.51320.82

[101] Oblak A , Jerala R . Toll‐like receptor 4 activation in cancer progression and therapy. Clin Dev Immunol. 2011;2011:609579. 22110526 10.1155/2011/609579 PMC3216292

[102] Parker KH , Horn LA , Ostrand‐Rosenberg S . High‐mobility group box protein 1 promotes the survival of myeloid‐derived suppressor cells by inducing autophagy. J Leukoc Biol. 2016;100(3):463‐470. 26864266 10.1189/jlb.3HI0715-305R PMC4982609

[103] Kayagaki N , Lee BL , Stowe IB , et al. IRF2 transcriptionally induces GSDMD expression for pyroptosis. Sci Signal. 2019;12(582). 10.1126/scisignal.aax4917 31113851

[104] Liu Y , Fang Y , Chen X , et al. Gasdermin E‐mediated target cell pyroptosis by CAR T cells triggers cytokine release syndrome. Sci Immunol. 2020;5(43). 10.1126/sciimmunol.aax7969 31953257

[105] Cao W , Chen G , Wu L , et al. Ionizing radiation triggers the antitumor immunity by inducing gasdermin E‐mediated pyroptosis in tumor cells. Int J Radiat Oncol Biol Phys. 2023;115(2):440‐452. 35918054 10.1016/j.ijrobp.2022.07.1841

[106] Zhang T , Li Y , Zhu R , et al. Transcription factor p53 suppresses tumor growth by prompting pyroptosis in non‐small‐cell lung cancer. Oxid Med Cell Longev. 2019;2019:8746895. 31737176 10.1155/2019/8746895 PMC6815571

[107] Gao J , Qiu X , Xi G , et al. Downregulation of GSDMD attenuates tumor proliferation via the intrinsic mitochondrial apoptotic pathway and inhibition of EGFR/Akt signaling and predicts a good prognosis in non‑small cell lung cancer. Oncol Rep. 2018;40(4):1971‐1984. 30106450 10.3892/or.2018.6634 PMC6111570

[108] Xi G , Gao J , Wan B , et al. GSDMD is required for effector CD8(+) T cell responses to lung cancer cells. Int Immunopharmacol. 2019;74:105713. 31276977 10.1016/j.intimp.2019.105713

[109] Wei J , Xu Z , Chen X , et al. Overexpression of GSDMC is a prognostic factor for predicting a poor outcome in lung adenocarcinoma. Mol Med Rep. 2020;21(1):360‐370. 31939622 10.3892/mmr.2019.10837 PMC6896373

[110] Lu C , Guo C , Chen H , et al. A novel chimeric PD1‐NKG2D‐41BB receptor enhances antitumor activity of NK92 cells against human lung cancer H1299 cells by triggering pyroptosis. Mol Immunol. 2020;122:200‐206. 32388482 10.1016/j.molimm.2020.04.016

[111] Peng Z , Wang P , Song W , et al. GSDME enhances cisplatin sensitivity to regress non‐small cell lung carcinoma by mediating pyroptosis to trigger antitumor immunocyte infiltration. Signal Transduct Target Ther. 2020;5(1):159. 32839451 10.1038/s41392-020-00274-9 PMC7445264

[112] Yuan R , Zhao W , Wang QQ , et al. Cucurbitacin B inhibits non‐small cell lung cancer in vivo and in vitro by triggering TLR4/NLRP3/GSDMD‐dependent pyroptosis. Pharmacol Res. 2021;170:105748. 34217831 10.1016/j.phrs.2021.105748

[113] Teng JF , Mei QB , Zhou XG , et al. Polyphyllin VI induces caspase‐1‐mediated pyroptosis via the induction of ROS/NF‐κB/NLRP3/GSDMD signal axis in non‐small cell lung cancer. Cancers (Basel). 2020;12(1). 10.3390/cancers12010193 PMC7017302 31941010

[114] Wang F , Liu W , Ning J , et al. Simvastatin suppresses proliferation and migration in non‐small cell lung cancer via pyroptosis. Int J Biol Sci. 2018;14(4):406‐417. 29725262 10.7150/ijbs.23542 PMC5930473

[115] Zhang J , Chen Y , He Q . Distinct characteristics of dasatinib‐induced pyroptosis in gasdermin E‐expressing human lung cancer A549 cells and neuroblastoma SH‐SY5Y cells. Oncol Lett. 2020;20(1):145‐154. 10.3892/ol.2020.11556 PMC7285962 32565942

[116] Lu H , Zhang S , Wu J , et al. Molecular targeted therapies elicit concurrent apoptotic and GSDME‐dependent pyroptotic tumor cell death. Clin Cancer Res. 2018;24(23):6066‐6077. 30061362 10.1158/1078-0432.CCR-18-1478

[117] Xu X , Zhou X , Chen Z , Gao C , Zhao L , Cui Y . Silencing of lncRNA XIST inhibits non‐small cell lung cancer growth and promotes chemosensitivity to cisplatin. Aging (Albany NY). 2020;12(6):4711‐4726. 10.18632/aging.102673. 32209729 10.18632/aging.102673 PMC7138551

[118] Liu J , Yao L , Zhang M , Jiang J , Yang M , Wang Y . Downregulation of LncRNA‐XIST inhibited development of non‐small cell lung cancer by activating miR‐335/SOD2/ROS signal pathway mediated pyroptotic cell death. Aging (Albany NY). 2019;11(18):7830‐7846. 10.18632/aging.102291. 31553952 10.18632/aging.102291 PMC6781979

[119] Shi F , Zhang L , Liu X , Wang Y . Knock‐down of microRNA miR‐556‐5p increases cisplatin‐sensitivity in non‐small cell lung cancer (NSCLC) via activating NLR family pyrin domain containing 3 (NLRP3)‐mediated pyroptotic cell death. Bioengineered. 2021;12(1):6332‐6342. 34488537 10.1080/21655979.2021.1971502 PMC8806686

[120] Xie B , Liu T , Chen S , et al. Combination of DNA demethylation and chemotherapy to trigger cell pyroptosis for inhalation treatment of lung cancer. Nanoscale. 2021;13(44):18608‐18615. 34730599 10.1039/d1nr05001j

[121] Jayabalan N , Oronsky B , Cabrales P , et al. A review of RRx‐001: a late‐stage multi‐indication inhibitor of NLRP3 activation and chronic inflammation. Drugs. 2023;83(5):389‐402. 36920652 10.1007/s40265-023-01838-z PMC10015535

[122] Morgensztern D , Rose M , Waqar SN , et al. RRx‐001 followed by platinum plus etoposide in patients with previously treated small‐cell lung cancer. Br J Cancer. 2019;121(3):211‐217. 31231122 10.1038/s41416-019-0504-8 PMC6738071

[123] Oronsky B , Abrouk N , Caroen S , et al. A 2022 update on extensive stage small‐cell lung cancer (SCLC). J Cancer. 2022;13(9):2945‐2953. 35912017 10.7150/jca.75622 PMC9330463

[124] Huang YL , Zhang GH , Zhu Q , Wu X , Wu LG . Expression levels of caspase‐3 and gasdermin E and their involvement in the occurrence and prognosis of lung cancer. Cancer Rep (Hoboken). 2022;5(9):e1561. 34553845 10.1002/cnr2.1561 PMC9458510

[125] Lin W , Chen Y , Wu B , Chen Y , Li Z . Identification of the pyroptosis‑related prognostic gene signature and the associated regulation axis in lung adenocarcinoma. Cell Death Discov. 2021;7(1):161. 34226539 10.1038/s41420-021-00557-2 PMC8257680

[126] Kim MS , Lebron C , Nagpal JK , et al. Methylation of the DFNA5 increases risk of lymph node metastasis in human breast cancer. Biochem Biophys Res Commun. 2008;370(1):38‐43. 18346456 10.1016/j.bbrc.2008.03.026 PMC3094717

[127] Wang Y , Peng J , Mi X , Yang M . p53‐GSDME elevation: a path for CDK7 inhibition to suppress breast cancer cell survival. Front Mol Biosci. 2021;8:697457. 34490348 10.3389/fmolb.2021.697457 PMC8417410

[128] Yan H , Luo B , Wu X , et al. Cisplatin induces pyroptosis via activation of MEG3/NLRP3/caspase‐1/GSDMD pathway in triple‐negative breast cancer. Int J Biol Sci. 2021;17(10):2606‐2621. 34326697 10.7150/ijbs.60292 PMC8315016

[129] Gong W , Fang P , Leng M , Shi Y . Promoting GSDME expression through DNA demethylation to increase chemosensitivity of breast cancer MCF‐7 /Taxol cells. PLoS One. 2023;18(3):e0282244. 36867605 10.1371/journal.pone.0282244 PMC9983855

[130] Zhang Z , Zhang H , Li D , Zhou X , Qin Q , Zhang Q . Caspase‐3‐mediated GSDME induced pyroptosis in breast cancer cells through the ROS/JNK signalling pathway. J Cell Mol Med. 2021;25(17):8159‐8168. 34369076 10.1111/jcmm.16574 PMC8419174

[131] Zheng Z , Bian Y , Zhang Y , Ren G , Li G . Metformin activates AMPK/SIRT1/NF‐κB pathway and induces mitochondrial dysfunction to drive caspase3/GSDME‐mediated cancer cell pyroptosis. Cell Cycle. 2020;19(10):1089‐1104. 32286137 10.1080/15384101.2020.1743911 PMC7217368

[132] Pizato N , Luzete BC , Kiffer L , et al. Omega‐3 docosahexaenoic acid induces pyroptosis cell death in triple‐negative breast cancer cells. Sci Rep. 2018;8(1):1952. 29386662 10.1038/s41598-018-20422-0 PMC5792438

[133] Li Y , Wang W , Li A , et al. Dihydroartemisinin induces pyroptosis by promoting the AIM2/caspase‐3/DFNA5 axis in breast cancer cells. Chem Biol Interact. 2021;340:109434. 33689708 10.1016/j.cbi.2021.109434

[134] Nohara K , Mallampalli V , Nemkov T , et al. Nobiletin fortifies mitochondrial respiration in skeletal muscle to promote healthy aging against metabolic challenge. Nat Commun. 2019;10(1):3923. 31462679 10.1038/s41467-019-11926-y PMC6713763

[135] Wang JG , Jian WJ , Li Y , Zhang J . Nobiletin promotes the pyroptosis of breast cancer via regulation of miR‐200b/JAZF1 axis. Kaohsiung J Med Sci. 2021;37(7):572‐582. 33728753 10.1002/kjm2.12371 PMC11896515

[136] Du QH , Peng C , Zhang H . Polydatin: a review of pharmacology and pharmacokinetics. Pharm Biol. 2013;51(11):1347‐1354. 23862567 10.3109/13880209.2013.792849

[137] Liu M , Li Y , Kong B , Zhang G , Zhang Q . Polydatin down‐regulates the phosphorylation level of STAT3 and induces pyroptosis in triple‐negative breast cancer mice with a high‐fat diet. Ann Transl Med. 2022;10(4):173. 10.21037/atm‐22‐73. 35280371 10.21037/atm-22-73 PMC8908165

[138] An H , Heo JS , Kim P , et al. Tetraarsenic hexoxide enhances generation of mitochondrial ROS to promote pyroptosis by inducing the activation of caspase‐3/GSDME in triple‐negative breast cancer cells. Cell Death Dis. 2021;12(2):159. 33558527 10.1038/s41419-021-03454-9 PMC7870965

[139] Elion DL , Jacobson ME , Hicks DJ , et al. Therapeutically active RIG‐I agonist induces immunogenic tumor cell killing in breast cancers. Cancer Res. 2018;78(21):6183‐6195. 30224377 10.1158/0008-5472.CAN-18-0730

[140] Tamura Y , Morikawa M , Tanabe R , Miyazono K , Koinuma D . Anti‐pyroptotic function of TGF‐β is suppressed by a synthetic dsRNA analogue in triple negative breast cancer cells. Mol Oncol. 2021;15(5):1289‐1307. 33342034 10.1002/1878-0261.12890 PMC8096786

[141] Aznar MA , Planelles L , Perez‐Olivares M , et al. Immunotherapeutic effects of intratumoral nanoplexed poly I:c. J Immunother Cancer. 2019;7(1):116. 31046839 10.1186/s40425-019-0568-2 PMC6498680

[142] Zhou H , He H , Liang R , et al. In situ poly I:c released from living cell drug nanocarriers for macrophage‐mediated antitumor immunotherapy. Biomaterials. 2021;269:120670. 33485214 10.1016/j.biomaterials.2021.120670

[143] Zhao P , Wang M , Chen M , et al. Programming cell pyroptosis with biomimetic nanoparticles for solid tumor immunotherapy. Biomaterials. 2020;254:120142. 32485591 10.1016/j.biomaterials.2020.120142

[144] Su W , Qiu W , Li SJ , et al. A dual‐responsive STAT3 inhibitor nanoprodrug combined with oncolytic virus elicits synergistic antitumor immune responses by igniting pyroptosis. Adv Mater. 2023;35(11):e2209379. 36545949 10.1002/adma.202209379

[145] Molina‐Crespo Á , Cadete A , Sarrio D , et al. Intracellular delivery of an antibody targeting gasdermin‐B reduces HER2 breast cancer aggressiveness. Clin Cancer Res. 2019;25(15):4846‐4858. 31064780 10.1158/1078-0432.CCR-18-2381

[146] Wu X , Mao X , Huang Y , Zhu Q , Guan J , Wu L . Detection of proteins associated with the pyroptosis signaling pathway in breast cancer tissues and their significance. Int J Clin Exp Pathol. 2020;13(6):1408‐1414. 32661477 PMC7344002

[147] Hergueta‐Redondo M , Sarrio D , Molina‐Crespo Á , et al. Gasdermin B expression predicts poor clinical outcome in HER2‐positive breast cancer. Oncotarget. 2016;7(35):56295‐56308. 10.18632/oncotarget.10787. 27462779 10.18632/oncotarget.10787 PMC5302915

[148] Croes L , de Beeck KO , Pauwels P , et al. DFNA5 promoter methylation a marker for breast tumorigenesis. Oncotarget. 2017;8(19):31948‐31958. 10.18632/oncotarget.16654. 28404884 10.18632/oncotarget.16654 PMC5458261

[149] Winer EP , Lipatov O , Im SA , et al. Pembrolizumab versus investigator‐choice chemotherapy for metastatic triple‐negative breast cancer (KEYNOTE‐119): a randomised, open‐label, phase 3 trial. Lancet Oncol. 2021;22(4):499‐511. 33676601 10.1016/S1470-2045(20)30754-3

[150] Salas‐Benito D , Pérez‐Gracia JL , Ponz‐Sarvisé M , et al. Paradigms on immunotherapy combinations with chemotherapy. Cancer Discov. 2021;11(6):1353‐1367. 33712487 10.1158/2159-8290.CD-20-1312

[151] López MJ , Carbajal J , Alfaro AL , et al. Characteristics of gastric cancer around the world. Crit Rev Oncol Hematol. 2023;181:103841. 36240980 10.1016/j.critrevonc.2022.103841

[152] Saeki N , Usui T , Aoyagi K , et al. Distinctive expression and function of four GSDM family genes (GSDMA‐D) in normal and malignant upper gastrointestinal epithelium. Genes Chromosomes Cancer. 2009;48(3):261‐271. 19051310 10.1002/gcc.20636

[153] Wang WJ , Chen D , Jiang MZ , et al. Downregulation of gasdermin D promotes gastric cancer proliferation by regulating cell cycle‐related proteins. J Dig Dis. 2018;19(2):74‐83. 29314754 10.1111/1751-2980.12576

[154] Komiyama H , Aoki A , Tanaka S , et al. Alu‐derived cis‐element regulates tumorigenesis‐dependent gastric expression of GASDERMIN B (GSDMB). Genes Genet Syst. 2010;85(1):75‐83. 20410667 10.1266/ggs.85.75

[155] Wang Y , Yin B , Li D , Wang G , Han X , Sun X . GSDME mediates caspase‐3‐dependent pyroptosis in gastric cancer. Biochem Biophys Res Commun. 2018;495(1):1418‐1425. 29183726 10.1016/j.bbrc.2017.11.156

[156] Fu L , Yonemura A , Yasuda‐Yoshihara N , et al. Intracellular MUC20 variant 2 maintains mitochondrial calcium homeostasis and enhances drug resistance in gastric cancer. Gastric Cancer. 2022;25(3):542‐557. 35166958 10.1007/s10120-022-01283-z

[157] Zhang X , Li C , Chen D , et al. pylori CagA activates the NLRP3 inflammasome to promote gastric cancer cell migration and invasion. Inflamm Res. 2022;71(1):141‐155. 34854954 10.1007/s00011-021-01522-6

[158] Li L , Bao B , Chai X , et al. The anti‐inflammatory effect of Callicarpa nudiflora extract on H. Pylori‐infected GES‐1 cells through the inhibition of ROS/NLRP3/caspase‐1/IL‐1β signaling axis. Can J Infect Dis Med Microbiol. 2022;2022:5469236. 35873363 10.1155/2022/5469236 PMC9307406

[159] Huang J , Fan P , Liu M , et al. Famotidine promotes inflammation by triggering cell pyroptosis in gastric cancer cells. BMC Pharmacol Toxicol. 2021;22(1):62. 34686215 10.1186/s40360-021-00533-7 PMC8539739

[160] Zhang F , Yin Y , Xu W , et al. Icariin inhibits gastric cancer cell growth by regulating the hsa_circ_0003159/miR‐223‐3p/NLRP3 signaling axis. Hum Exp Toxicol. 2022;41:9603271221097363. 35532261 10.1177/09603271221097363

[161] Xia Y , Jin Y , Cui D , et al. Antitumor effect of simvastatin in combination with DNA methyltransferase inhibitor on gastric cancer via. Front Pharmacol. 2022;13:860546. 35517821 10.3389/fphar.2022.860546 PMC9065610

[162] Wang JM , Sheng YC , Ji LL , Wang ZT . Ferulic acid prevents liver injury and increases the anti‐tumor effect of diosbulbin B in vivo. J Zhejiang Univ Sci B. 2014;15(6):540‐547. 24903991 10.1631/jzus.B1300250 PMC4116857

[163] Li C , Qiu J , Xue Y . Low‐dose Diosbulbin‐B (DB) activates tumor‐intrinsic PD‐L1/NLRP3 signaling pathway mediated pyroptotic cell death to increase cisplatin‐sensitivity in gastric cancer (GC). Cell Biosci. 2021;11(1):38. 33579380 10.1186/s13578-021-00548-x PMC7881658

[164] Deng BB , Jiao BP , Liu YJ , Li YR , Wang GJ . BIX‐01294 enhanced chemotherapy effect in gastric cancer by inducing GSDME‐mediated pyroptosis. Cell Biol Int. 2020;44(9):1890‐1899. 32437063 10.1002/cbin.11395 PMC7496303

[165] Ren N , Jiang T , Wang C , et al. LncRNA ADAMTS9‐AS2 inhibits gastric cancer (GC) development and sensitizes chemoresistant GC cells to cisplatin by regulating miR‐223‐3p/NLRP3 axis. Aging (Albany NY). 2020;12(11):11025‐11041. 10.18632/aging.103314. 32516127 10.18632/aging.103314 PMC7346038

[166] Long Y , Wang Z , Fan J , et al. A hybrid membrane coating nanodrug system against gastric cancer via the VEGFR2/STAT3 signaling pathway. J Mater Chem B. 2021;9(18):3838‐3855. 33908580 10.1039/d1tb00029b

[167] Yu Z , Cao W , Han C , et al. Biomimetic metal‐organic framework nanoparticles for synergistic combining of SDT‐chemotherapy induce pyroptosis in gastric cancer. Front Bioeng Biotechnol. 2022;10:796820. 35265591 10.3389/fbioe.2022.796820 PMC8899015

[168] Wang Z , Cao L , Zhou S , Lyu J , Gao Y , Yang R . Construction and validation of a novel pyroptosis‐related Four‐lncRNA prognostic signature related to gastric cancer and immune infiltration. Front Immunol. 2022;13:854785. 35392086 10.3389/fimmu.2022.854785 PMC8980360

[169] Liang C , Fan J , Liang C , Guo J . Identification and validation of a pyroptosis‐related prognostic model for gastric cancer. Front Genet. 2021;12:699503. 35280928 10.3389/fgene.2021.699503 PMC8916103

[170] Arnold M , Sierra MS , Laversanne M , Soerjomataram I , Jemal A , Bray F . Global patterns and trends in colorectal cancer incidence and mortality. Gut. 2017;66(4):683‐691. 26818619 10.1136/gutjnl-2015-310912

[171] Eaden JA , Abrams KR , Mayberry JF . The risk of colorectal cancer in ulcerative colitis: a meta‐analysis. Gut. 2001;48(4):526‐535. 11247898 10.1136/gut.48.4.526 PMC1728259

[172] Allen IC , TeKippe EM , Woodford RM , et al. The NLRP3 inflammasome functions as a negative regulator of tumorigenesis during colitis‐associated cancer. J Exp Med. 2010;207(5):1045‐1056. 20385749 10.1084/jem.20100050 PMC2867287

[173] Hansen JM , de Jong MF , Wu Q , et al. Pathogenic ubiquitination of GSDMB inhibits NK cell bactericidal functions. Cell. 2021;184(12):3178‐3191. 34022140 10.1016/j.cell.2021.04.036 PMC8221529

[174] Liu Z , Li Y , Zhu Y , et al. Apoptin induces pyroptosis of colorectal cancer cells via the GSDME‐dependent pathway. Int J Biol Sci. 2022;18(2):717‐730. 35002520 10.7150/ijbs.64350 PMC8741846

[175] Ma Y , Chen Y , Lin C , Hu G . Biological functions and clinical significance of the newly identified long non‑coding RNA RP1‑85F18.6 in colorectal cancer. Oncol Rep. 2018;40(5):2648‐2658. 30226619 10.3892/or.2018.6694 PMC6151894

[176] Wu LS , Liu Y , Wang XW , et al. LPS enhances the chemosensitivity of oxaliplatin in HT29 cells via GSDMD‐mediated pyroptosis. Cancer Manag Res. 2020;12:10397‐10409. 33116894 10.2147/CMAR.S244374 PMC7585788

[177] Yu J , Li S , Qi J , et al. Cleavage of GSDME by caspase‐3 determines lobaplatin‐induced pyroptosis in colon cancer cells. Cell Death Dis. 2019;10(3):193. 30804337 10.1038/s41419-019-1441-4 PMC6389936

[178] Chen C , Wang B , Sun J , et al. DAC can restore expression of NALP1 to suppress tumor growth in colon cancer. Cell Death Dis. 2015;6(1):e1602. 25611377 10.1038/cddis.2014.532 PMC4669739

[179] Chen T , Wang Z , Zhong J , et al. Secoisolariciresinol diglucoside induces pyroptosis by activating caspase‐1 to cleave GSDMD in colorectal cancer cells. Drug Dev Res. 2022;83(5):1152‐1166. 35472101 10.1002/ddr.21939 PMC9543314

[180] Tian W , Wang Z , Tang NN , et al. Ascorbic acid sensitizes colorectal carcinoma to the cytotoxicity of arsenic trioxide via promoting reactive oxygen species‐dependent apoptosis and pyroptosis. Front Pharmacol. 2020;11:123. 32153415 10.3389/fphar.2020.00123 PMC7047232

[181] Guo J , Zheng J , Mu M , et al. GW4064 enhances the chemosensitivity of colorectal cancer to oxaliplatin by inducing pyroptosis. Biochem Biophys Res Commun. 2021;548:60‐66. 33631675 10.1016/j.bbrc.2021.02.043

[182] Sala R , Rioja‐Blanco E , Serna N , et al. GSDMD‐dependent pyroptotic induction by a multivalent CXCR4‐targeted nanotoxin blocks colorectal cancer metastases. Drug Deliv. 2022;29(1):1384‐1397. 35532120 10.1080/10717544.2022.2069302 PMC9090371

[183] Serna N , Álamo P , Ramesh P , et al. Nanostructured toxins for the selective destruction of drug‐resistant human CXCR4(+) colorectal cancer stem cells. J Control Release. 2020;320:96‐104. 31931052 10.1016/j.jconrel.2020.01.019

[184] Fan JX , Deng RH , Wang H , et al. Epigenetics‐based tumor cells pyroptosis for enhancing the immunological effect of chemotherapeutic nanocarriers. Nano Lett. 2019;19(11):8049‐8058. 31558023 10.1021/acs.nanolett.9b03245

[185] Lim MC , Chang SJ , Park B , et al. Survival after hyperthermic intraperitoneal chemotherapy and primary or interval cytoreductive surgery in ovarian cancer: a randomized clinical trial. JAMA Surg. 2022;157(5):374‐383. 35262624 10.1001/jamasurg.2022.0143 PMC8908225

[186] Gu B , Shang X , Yan M , et al. Variations in incidence and mortality rates of endometrial cancer at the global, regional, and national levels, 1990–2019. Gynecol Oncol;161(2):573‐580. 10.1016/j.ygyno.2021.01.036 33551200

[187] Yang X , Wang J . The role of metabolic syndrome in endometrial cancer: a review. Front Oncol. 2019;9:744. 31440472 10.3389/fonc.2019.00744 PMC6694738

[188] Hipólito A , Martins F , Mendes C , Lopes‐Coelho F , Serpa J . Molecular and metabolic reprogramming: pulling the strings toward tumor metastasis. Front Oncol. 2021;11:656851. 34150624 10.3389/fonc.2021.656851 PMC8209414

[189] Guo J , Ye F , Xie W , et al. The HOXC‐AS2/miR‐876‐5p/HKDC1 axis regulates endometrial cancer progression in a high glucose‐related tumor microenvironment. Cancer Sci. 2022;113(7):2297‐2310. 35485648 10.1111/cas.15384 PMC9277262

[190] Liu SG , Wu XX , Hua T , et al. NLRP3 inflammasome activation by estrogen promotes the progression of human endometrial cancer. Onco Targets Ther. 2019;12:6927‐6936. 31695408 10.2147/OTT.S218240 PMC6717726

[191] Yang Y , Liu PY , Bao W , Chen SJ , Wu FS , Zhu PY . Hydrogen inhibits endometrial cancer growth via a ROS/NLRP3/caspase‐1/GSDMD‐mediated pyroptotic pathway. BMC Cancer. 2020;20(1):28. 31924176 10.1186/s12885-019-6491-6 PMC6954594

[192] Liang D , Hu M , Tang Q , Huang M , Tang L . Nine pyroptosis‐related lncRNAs are identified as biomarkers for predicting the prognosis and immunotherapy of endometrial carcinoma. Int J Gen Med. 2021;14:8073‐8085. 34803394 10.2147/IJGM.S338298 PMC8594792

[193] Zhang X , Yang Q . A pyroptosis‐related gene panel in prognosis prediction and immune microenvironment of human endometrial cancer. Front Cell Dev Biol. 2021;9:705828. 34722500 10.3389/fcell.2021.705828 PMC8551636

[194] Bray F , Ferlay J , Soerjomataram I , Siegel RL , Torre LA , Jemal A . Global cancer statistics 2018: gLOBOCAN estimates of incidence and mortality worldwide for 36 cancers in 185 countries. CA Cancer J Clin;68(6):394‐424. 30207593 10.3322/caac.21492

[195] Ma X , Yang M . The correlation between high‐risk HPV infection and precancerous lesions and cervical cancer. Am J Transl Res. 2021;13(9):10830‐10836. 34650762 PMC8507010

[196] So D , Shin HW , Kim J , et al. Cervical cancer is addicted to SIRT1 disarming the AIM2 antiviral defense. Oncogene. 2018;37(38):5191‐5204. 29844574 10.1038/s41388-018-0339-4

[197] Cho YS , Kang JW , Cho M , et al. Down modulation of IL‐18 expression by human papillomavirus type 16 E6 oncogene via binding to IL‐18. FEBS Lett. 2001;501(2‐3):139‐145. 11470273 10.1016/s0014-5793(01)02652-7

[198] Chen J , Ge L , Shi X , et al. Lobaplatin induces pyroptosis in cervical cancer cells via the caspase‐3/GSDME pathway. Anticancer Agents Med Chem. 2022;22(11):2091‐2097. 34666646 10.2174/1871520621666211018100532

[199] Chen D , Guo S , Tang X , et al. Combination of ruthenium (II) polypyridyl complex Δ‐Ru1 and Taxol enhances the anti‐cancer effect on Taxol‐resistant cancer cells through Caspase‐1/GSDMD‐mediated pyroptosis. J Inorg Biochem. 2022;230:111749. 35144218 10.1016/j.jinorgbio.2022.111749

[200] Xie J , Liu J , Liu H , et al. The antitumor effect of tanshinone IIA on anti‐proliferation and decreasing VEGF/VEGFR2 expression on the human non‐small cell lung cancer A549 cell line. Acta Pharm Sin B. 2015;5(6):554‐563. 26713270 10.1016/j.apsb.2015.07.008 PMC4675810

[201] Tong W , Guo J , Yang C . Tanshinone II A enhances pyroptosis and represses cell proliferation of HeLa cells by regulating miR‐145/GSDMD signaling pathway. Biosci Rep. 2020;40(4). 10.1042/BSR20200259 PMC7160242 32232409

[202] Yu S , Zhao N , He M , Zhang K , Bi X . MiRNA‐214 promotes the pyroptosis and inhibits the proliferation of cervical cancer cells via regulating the expression of NLRP3. Cell Mol Biol (Noisy‐le‐grand). 2020;66(6):59‐64. 33040786

[203] Hu H , Yang M , Dong W , et al. A pyroptosis‐related gene panel for predicting the prognosis and immune microenvironment of cervical cancer. Front Oncol. 2022;12:873725. 35574296 10.3389/fonc.2022.873725 PMC9099437

[204] Gaona‐Luviano P , Medina‐Gaona LA , Magaña‐Pérez K , Epidemiology of ovarian cancer. Chinese Clinical Oncology. 2020;9(4):47. 32648448 10.21037/cco-20-34

[205] Berkel C , Cacan E . Lower expression of NINJ1 (Ninjurin 1), a mediator of plasma membrane rupture, is associated with advanced disease and worse prognosis in serous ovarian cancer. Immunol Res. 2023;71(1):15‐28. 36184655 10.1007/s12026-022-09323-7

[206] Berkel C , Cacan E . Differential expression and copy number variation of gasdermin (GSDM) family members, pore‐forming proteins in pyroptosis, in normal and malignant serous ovarian tissue. Inflammation. 2021;44(6):2203‐2216. 34091823 10.1007/s10753-021-01493-0

[207] Mulvihill E , Sborgi L , Mari SA , Pfreundschuh M , Hiller S , Müller DJ . Mechanism of membrane pore formation by human gasdermin‐D. EMBO J. 2018;37(14). 10.15252/embj.201798321. 10.15252/embj.201798321 PMC6043855 29898893

[208] Szlasa W , Zendran I , Zalesińska A , Tarek M , Kulbacka J . Lipid composition of the cancer cell membrane. J Bioenerg Biomembr. 2020;52(5):321‐342. 32715369 10.1007/s10863-020-09846-4 PMC7520422

[209] He J , Siu MKY , Ngan HYS , Chan KKL . Aberrant cholesterol metabolism in ovarian cancer: identification of novel therapeutic targets. Front Oncol. 2021;11:738177. 34820325 10.3389/fonc.2021.738177 PMC8606538

[210] Li BY , Mohanraj D , Olson MC , et al. Human ovarian epithelial cancer cells cultures in vitro express both interleukin 1 alpha and beta genes. Cancer Res. 1992;52(8):2248‐2252. 1559228

[211] Woolery KT , Mohamed M , Linger RJ , Dobrinski KP , Roman J , Kruk PA . BRCA1 185delAG mutation enhances interleukin‐1β expression in ovarian surface epithelial cells. Biomed Res Int. 2015;2015:652017. 26357657 10.1155/2015/652017 PMC4556869

[212] Xu JF , Cen YX , Tang SS , Ren Y , Lyu WG . IL‐1β inhibitor sensitizes to olaparib in homologous recombination deficiency proficient ovarian cancer cells. Zhonghua Fu Chan Ke Za Zhi. 2022;57(7):519‐529. 35902786 10.3760/cma.j.cn112141-20220509-00307

[213] Uppendahl LD , Felices M , Bendzick L , et al. Cytokine‐induced memory‐like natural killer cells have enhanced function, proliferation, and in vivo expansion against ovarian cancer cells. Gynecol Oncol. 2019;153(1):149‐157. 30658847 10.1016/j.ygyno.2019.01.006 PMC6430659

[214] Simpkins F , Flores A , Chu C , et al. Chemoimmunotherapy using pegylated liposomal doxorubicin and interleukin‐18 in recurrent ovarian cancer: a phase I dose‐escalation study. Cancer Immunol Res. 2013;1(3):168‐178. 24777679 10.1158/2326-6066.CIR-13-0098

[215] Alrashed MM , Alharbi H , Alshehry AS , Ahmad M , Aloahd MS . MiR‐624‐5p enhances NLRP3 augmented gemcitabine resistance via EMT/IL‐1β/Wnt/β‐catenin signaling pathway in ovarian cancer. J Reprod Immunol. 2022;150:103488. 35124344 10.1016/j.jri.2022.103488

[216] Heath O , Berlato C , Maniati E , et al. Chemotherapy induces tumor‐associated macrophages that aid adaptive immune responses in ovarian cancer. Cancer Immunol Res. 2021;9(6):665‐681. 33839687 10.1158/2326-6066.CIR-20-0968 PMC7611478

[217] Tan C , Liu W , Zheng ZH , Wan XG . LncRNA HOTTIP inhibits cell pyroptosis by targeting miR‐148a‐3p/AKT2 axis in ovarian cancer. Cell Biol Int. 2021;45(7):1487‐1497. 33710684 10.1002/cbin.11588

[218] Li J , Yang C , Li Y , Chen A , Li L , You Z . LncRNA GAS5 suppresses ovarian cancer by inducing inflammasome formation. Biosci Rep. 2018;38(2). 10.1042/BSR20171150 PMC5857912 29229673

[219] Cao X , Zhang Q , Zhu Y , Huo X , Bao J , Su M . Derivation, comprehensive analysis, and assay validation of a pyroptosis‐related lncRNA prognostic signature in patients with ovarian cancer. Front Oncol. 2022;12:780950. 35280739 10.3389/fonc.2022.780950 PMC8912994

[220] Li J , Zhou L , Jiang H , Lin L , Li Y . Inhibition of FOSL2 aggravates the apoptosis of ovarian cancer cells by promoting the formation of inflammasomes. Genes Genomics. 2022;44(1):29‐38. 34773569 10.1007/s13258-021-01152-6 PMC8727396

[221] Boehm MF , Zhang L , Badea BA , et al. Synthesis and structure‐activity relationships of novel retinoid X receptor‐selective retinoids. J Med Chem. 1994;37(18):2930‐2941. 8071941 10.1021/jm00044a014

[222] Kobayashi T , Mitsuhashi A , Hongying P , et al. Bexarotene‐induced cell death in ovarian cancer cells through Caspase‐4‐gasdermin E mediated pyroptosis. Sci Rep. 2022;12(1):11123. 35778597 10.1038/s41598-022-15348-7 PMC9249775

[223] Zhang R , Chen J , Mao L , et al. Nobiletin triggers reactive oxygen species‐mediated pyroptosis through regulating autophagy in ovarian cancer cells. J Agric Food Chem. 2020;68(5):1326‐1336. 31955565 10.1021/acs.jafc.9b07908

[224] Kordulewska NK , Kostyra E , Cieślińska A , Matysiewicz M , Fiedorowicz E , Sienkiewicz‐Szłapka E . Changes in gene expression induced by histamine, fexofenadine and osthole: expression of histamine H(1) receptor, COX‐2, NF‐κB, CCR1, chemokine CCL5/RANTES and interleukin‐1β in PBMC allergic and non‐allergic patients. Immunobiology. 2017;222(3):571‐581. 27843000 10.1016/j.imbio.2016.11.004

[225] Bae H , Lee JY , Song J , Song G , Lim W . Osthole interacts with an ER‐mitochondria axis and facilitates tumor suppression in ovarian cancer. J Cell Physiol. 2021;236(2):1025‐1042. 32697363 10.1002/jcp.29913

[226] Sastry BV , Jaiswal N , Owens LK , Janson VE , Moore RD . 2‐(alpha‐Naphthoyl)ethyltrimethylammonium iodide and its beta‐isomer: new selective, stable and fluorescent inhibitors of choline acetyltransferase. J Pharmacol Exp Ther. 1988;245(1):72‐80. 3361452

[227] Qiao L , Wu X , Zhang J , et al. alpha‐NETA induces pyroptosis of epithelial ovarian cancer cells through the GSDMD/caspase‐4 pathway. FASEB J. 2019;33(11):12760‐12767. 31480859 10.1096/fj.201900483RR

[228] Wang X , Yin Y , Qian W , et al. Citric acid of ovarian cancer metabolite induces pyroptosis via the caspase‐4/TXNIP‐NLRP3‐GSDMD pathway in ovarian cancer. FASEB J. 2022;36(6):e22362. 35608902 10.1096/fj.202101993RR

[229] Hsu PC , Chao TK , Chou YC , et al. AIM2 inflammasome in tumor cells as a biomarker for predicting the treatment response to antiangiogenic therapy in epithelial ovarian cancer patients. J Clin Med. 2021;10(19). 10.3390/jcm10194529 PMC8509490 34640548

[230] Ding Y , Yan Y , Dong Y , et al. NLRP3 promotes immune escape by regulating immune checkpoints: a pan‐cancer analysis. Int Immunopharmacol. 2022;104:108512. 35026655 10.1016/j.intimp.2021.108512

[231] Ye Y , Dai Q , Qi H . A novel defined pyroptosis‐related gene signature for predicting the prognosis of ovarian cancer. Cell Death Discov. 2021;7(1):71. 33828074 10.1038/s41420-021-00451-x PMC8026591

[232] Gao L , Ying F , Cai J , et al. Identification and validation of pyroptosis‐related gene landscape in prognosis and immunotherapy of ovarian cancer. J Ovarian Res. 2023;16(1):27. 36707884 10.1186/s13048-022-01065-2 PMC9883900

[233] Li Y , Zheng JY , Liu JQ , et al. Succinate/NLRP3 inflammasome induces synovial fibroblast activation: therapeutical effects of clematichinenoside AR on arthritis. Front Immunol. 2016;7:532. 28003810 10.3389/fimmu.2016.00532 PMC5141240

[234] Tan G , Huang C , Chen J , Chen B , Zhi F . Gasdermin‐E‐mediated pyroptosis participates in the pathogenesis of Crohn's disease by promoting intestinal inflammation. Cell Rep. 2021;35(11):109265. 34133932 10.1016/j.celrep.2021.109265

[235] Kanai T , Watanabe M , Okazawa A , et al. Interleukin 18 is a potent proliferative factor for intestinal mucosal lymphocytes in Crohn's disease. Gastroenterology. 2000;119(6):1514‐1523. 11113073 10.1053/gast.2000.20260

[236] Shi X , Xie WL , Kong WW , Chen D , Qu P . Expression of the NLRP3 inflammasome in carotid atherosclerosis. J Stroke Cerebrovasc Dis. 2015;24(11):2455‐2466. 26381780 10.1016/j.jstrokecerebrovasdis.2015.03.024

[237] Luo B , Li B , Wang W , et al. NLRP3 gene silencing ameliorates diabetic cardiomyopathy in a type 2 diabetes rat model. PLoS One. 2014;9(8):e104771. 25136835 10.1371/journal.pone.0104771 PMC4138036

[238] Benjamin EJ , Blaha MJ , Chiuve SE , et al. Heart disease and stroke statistics‐2017 update: a report from the American Heart Association. Circulation. 2017;135(10):e146‐e603. 28122885 10.1161/CIR.0000000000000485 PMC5408160

[239] Lee JH , Kim HJ , Kim JU , et al. A novel treatment strategy by natural products in NLRP3 inflammasome‐mediated neuroinflammation in Alzheimer's and Parkinson's disease. Int J Mol Sci. 2021;22(3). 10.3390/ijms22031324 PMC7866084 33525754

[240] Nakanishi K , Yoshimoto T , Tsutsui H , Okamura H . Interleukin‐18 regulates both Th1 and Th2 responses. Annu Rev Immunol. 2001;19:423‐474. 11244043 10.1146/annurev.immunol.19.1.423

[241] Fabbi M , Carbotti G , Ferrini S . Context‐dependent role of IL‐18 in cancer biology and counter‐regulation by IL‐18BP. J Leukoc Biol. 2015;97(4):665‐675. 25548255 10.1189/jlb.5RU0714-360RR

[242] Ludwiczek O , Kaser A , Koch RO , Vogel W , Cruikshank WW , Tilg H . Activation of caspase‐3 by interferon alpha causes interleukin‐16 secretion but fails to modulate activation induced cell death. Eur Cytokine Netw. 2001;12(3):478‐486. 11566629

[243] Browning L , Patel MR , Horvath EB , Tawara K , Jorcyk CL . IL‐6 and ovarian cancer: inflammatory cytokines in promotion of metastasis. Cancer Manag Res. 2018;10:6685‐6693. 30584363 10.2147/CMAR.S179189 PMC6287645

[244] Karki R , Sharma BR , Tuladhar S , et al. Synergism of TNF‐α and IFN‐γ triggers inflammatory cell death, tissue damage, and mortality in SARS‐CoV‐2 infection and cytokine shock syndromes. Cell. 2021;184(1):149‐168. e17 33278357 10.1016/j.cell.2020.11.025 PMC7674074

[245] Coll RC , Robertson AA , Chae JJ , et al. A small‐molecule inhibitor of the NLRP3 inflammasome for the treatment of inflammatory diseases. Nat Med. 2015;21(3):248‐255. 25686105 10.1038/nm.3806 PMC4392179

[246] Coll RC , Hill JR , Day CJ , et al. MCC950 directly targets the NLRP3 ATP‐hydrolysis motif for inflammasome inhibition. Nat Chem Biol. 2019;15(6):556‐559. 31086327 10.1038/s41589-019-0277-7

[247] Martín‐Sánchez F , Diamond C , Zeitler M , et al. Inflammasome‐dependent IL‐1β release depends upon membrane permeabilisation. Cell Death Differ. 2016;23(7):1219‐1231. 26868913 10.1038/cdd.2015.176 PMC4946890

